# A Nanobody/Monoclonal Antibody “hybrid” sandwich technology offers an improved immunoassay strategy for detection of African trypanosome infections

**DOI:** 10.1371/journal.pntd.0012294

**Published:** 2024-07-01

**Authors:** Steven Odongo, Bo-Kyung Jin, Hang Thi Thu Nguyen, Magdalena Radwanska, Stefan Magez

**Affiliations:** 1 Laboratory for Biomedical Research, Department of Molecular Biotechnology, Environment Technology and Food Technology KR01, Ghent University Global Campus, Incheon, South Korea; 2 Department of Biomedical Molecular Biology, WE14, Ghent University, Ghent, Belgium; 3 Laboratory of Cellular and Molecular Immunology, Vrije Universiteit Brussel, Brussels, Belgium; 4 Department of Biochemistry and Microbiology, WE10, Ghent University, Ghent, Belgium; US Food and Drug Administration, UNITED STATES

## Abstract

The scarcity of reliable devices for diagnosis of Animal African trypanosomiasis (AAT) presents a limitation to control of the disease. Existing high-sensitivity technologies such as PCR are costly, laborious, time-consuming, complex, and require skilled personnel. Hence, utilisation of most diagnostics for AAT is impracticable in rural areas, where the disease occurs. A more accessible point-of-care test (POCT) capable of detecting cryptic active infection, without relying on expensive equipment, would facilitate AAT detection. In turn, early management, would reduce disease incidence and severity. Today, several ongoing research projects aim at modifying complex immunoassays into POCTs. In this context, we report the development of an antigen (Ag) detection sandwich ELISA prototype for diagnosis of *T*. *congolense* infections, which is comprised of nanobody (Nb) and monoclonal antibody (mAb) reagents.

The Nb474H used here, originated from a past study. Briefly, the Nb was engineered starting from mRNA of peripheral blood lymphocytes of an alpaca immunized with soluble lysate of *Trypanosoma congolense* (TC13). *T*. *congolense* glycosomal fructose-1,6-bisphosphate aldolase (*Tco*ALD) was discovered as the cognate Ag of Nb474H. In this study, splenocytes were harvested from a mouse immunized with recombinant *Tco*ALD and fused with NS01 cells to generate a hybridoma library. Random screening of the library on *Tco*ALD retrieved a lone binder, designated IgM8A2. Using Nb474H as Ag-capture reagent in combination with the IgM8A2 monoclonal antibody Ag-detection reagent resulted in a tool that effectively detects native *Tco*ALD released during infection by *T*. *congolense* parasites.

Hitherto, development of POCT for detection of active trypanosome infection is elusive. The Nanobody/Monoclonal Antibody (Nb/mAb) “hybrid” sandwich technology offers prospects for exploration, using the unique specificity of Nb as a key determinant in Ag capturing, while using the versatility of monoclonal Ab to adapt to various detection conditions.

## Author summary

Animal African trypanosomiasis is a parasitic wasting disease, which mainly affects livestock of poor households in remote rural areas of sub-Saharan Africa, Asia and Latin America. Affordable interventions are primary to eradication campaigns. The latter is mandated to the Pan African Tsetse and Trypanosomiasis Eradication Campaign (PATTEC). PATTEC conducts activities including mass screening of human population for Human African trypanosomiasis (HAT) and treating positive cases. This strategy has dramatically reduced cases of HAT over the last two decades. Highly sensitive and specific current tests for AAT are technically demanding, confining their use to well-equipped reference laboratories in urban centres. Inaccessibility of tests for AAT by the locals living in remote areas, hinders case finding and delays treatment intervention, eventually perpetuating disease transmission and severity. Hence, the aim of our ongoing work is to ultimately convert a complex laboratory-based test for AAT caused by *T*. *congolense*, developed earlier by our research team, into a simple hand-held device suitable for use in the field. The current test format employs a nanobody-monoclonal antibody sandwich combination to detect *T*. *congolense* infections, through detection of trypanosome Fructose-1,6-bisphosphate aldolase that is present in the blood of infected animals. The diagnostic capability of the assay was successfully demonstrated on experimental samples.

## Introduction

African trypanosomiasis (AT) is a devastating disease of humans and animals. Elimination of Human African Trypanosomiasis (HAT) of sleeping sickness, as a public health threat, is being targeted by the year 2030 [1]. The realization of this elimination would be a milestone in the fulfilment of the United Nations’s Sustainable Development Goals of eradicating poverty, ending hunger, and promoting good-health and well-being of the people [2]. Persistence of AT has affected development in communities where the disease occurs. Whereas trypanosome species responsible for HAT are restricted to Africa, some of the species causing the Animal African Trypanosomiasis (AAT) are prevalent far beyond the borders of Africa [3,4]. Hence, AT cases have been reported in South America [5], the Mediterranean Europe [6], and Asia [7,8] qualifying it among the most widely spread animal diseases in the world. Generally, AT is responsible for direct loss of lives [9,10], and it has a negative impact on the economy [11]. Employing chemicals to control the tsetse fly vector for AT, has raised environmental concerns including indiscriminate killing of non-target animals [12]. While elimination of AT would relieve the affected communities from the disease burden, it is both complex and highly demanding. An integrated approach involving vector control, case detection and treatment, has seen a near-elimination of *gambiense*-HAT cases in the disease endemic regions [13] holding promise for a future elimination altogether.

Deployment of accessible low-cost diagnostics that readily reveal trypanosomes in the host, as well as reservoir species, would be valuable for guiding treatment decision, monitoring control program and surveillance. Unfortunately, current tests for trypanosomiasis do not meet the ASSURED criteria required by the World Health Organization [14]. Largely, the existing tests for trypanosomes are costly, less sensitive, less specific, inherently complex, laborious and time-consuming, require qualified personnel, and rely on electricity making them unsuitable for use in resource-deprived rural communities. Deployment of reliable point-of-care tests (POCT) [15] for AAT on farms, would enable case finding and allow prompt treatment intervention. The only field POCT for AAT are card agglutination test (CATT/*T*. *evansi*) for detection of *T*. *evansi* [16], and *VerY Diag* (CEVA), which is a Lateral Flow Assay (LFA) for multiplex detection of *T*. *congolense* and *T*. *vivax* species. While these tests have aided epidemiological investigations of the target trypanosome species, they are Ab detection tests incapable of differentiating active infections from past exposures, hence restricting their scope. Where the test result is needed to inform treatment decisions, deployment of a confirmatory test is crucial. Microscopy and polymerase chain reaction (PCR) are valuable tests employed by reference laboratories to confirm AAT. However, microscopy is ineffective when specimen examination is delayed, time-consuming, laborious, not always field adapted, and it requires technical expertise. PCR on the other hand, is a high throughput sensitivity test; however, the technique requires reliable access to electricity and it is technically demanding. Therefore, conventional PCRs are not ideally suited for field operations. In contrast, Ag detection tests are amiable tool of choice for development of POCT for trypanosomiases, because they confirm active infection as well as drug failure. The deployment of Ag detection POCT has facilitated the control of malaria [17] and COVID-19 [18] among other infectious diseases. However, past efforts to develop similar mAb-based Ag detection tests for trypanosomiasis have been futile [19]. The mAb-based Ag detection test prototype for trypanosomiasis suffers from low sensitivity, mostly attributed to low Ag loads in specimens resulting from sequestration of Ag in immune complex, and an inherently low pathogen loads [20]. The first description of Nb technology 30 years ago by Hamers-Casterman *et al* [21] reinvigorated discovery research, targeting the technology, for the development of Ag detection tests for AAT [22,23]. Typically, Nbs possess a relatively extended complementarity determining region three (CDR3), compared to conventional Abs [24], thus allowing their preferential bindings to cryptic epitopes [25,26]. The fact that Nbs can bind Ag-Ab immune complexes through a unique epitope recognition, motivated its exploration as a tool of choice for outwitting low sensitivity of Ag-detection tests, caused by parasite-induced host Abs interference.

Whereas developments of several Nb-based diagnostic devices for trypanosomiasis were initiated [22,23,27], commercialization of these tests has not yet been achieved for practical reasons. Firstly, some of these Nb-based devices are detecting their respective target Ags in a homologous sandwich fashion [22] making translation into an Ag capture LFA without loss of sensitivity impossible. Indeed, in an Ag detection LFA, the target Ag is pre-complexed with high amounts of detection Nbs and the complex is driven by capillary action to a line of printed Ag-capture Nbs. In a homologous sandwich format, the Ag-capture Nbs would compete for the same binding sites with the already-couped detection Nb. In this case, most of the complexes would escape capture, causing a drastic reduction in signal intensity and consequently lowering the assay sensitivity. A second limitation affecting development of Nb-based test devices is the small size of Nbs, which often compromise conjugation to Gold (*Au*) nanoparticles [28]. To mitigate these limitations, but still exploit the highly specific Nb-capturing capacity, we explored here a Nb/mAb “hybrid” heterologous sandwich system. As our previous research has shown that trypanosome aldolase is a target that allows for a highly sensitive detection of active trypanosome infection [22], the target of the new test format was kept the same. Hence, a mouse mAb (IgM8A2) was generated against *Tco*ALD and integrated in the ELISA to substitute the previously described Nb detection reagent, thereby achieving a Nb474H/IgM8A2 “hybrid” sandwich setup. The capability of the “hybrid” sandwich system to detect native *Tco*ALD was demonstrated.

## Methods

### Ethics statement

Permission for the use of mice was granted by the Ghent University Global Campus Institutional Animal Use and Care committee (Project number: IACUC 2022–014).

### Mice

Eight-weeks old female mice BALB/c and C57BL/6N were procured from the Korean Animal Technology (KOATEC) Co. Ltd, Republic of Korea. The animals were acclimatized for a fortnight in a facility at the Biomedical Research Centre, the Ghent University Global Campus, Republic of Korea. While the BALB/c mice were used for generation of hybridoma, C57BL/N6 were used for culturing trypanosomes.

### Trypanosomes, trypanosome lysate, and sera

Trypanosomes used in the study were *Trypanosoma congolense* TC13, *T*. *b*. *brucei* An Tat 1.1E, *T*. *vivax* ILRAD 700, and *T*. *evansi* STIB 816. *Trypanosoma congolense* was propagated, purified, and homogenized into lysate according to the protocol described elsewhere [22]. Briefly, aliquots of frozen trypanosome stocks (50 μl) were each reconstituted in 1xPBS (500 μl). The viability of cells was checked by wet smear and live parasites were quantified by a haemocytometer (*Improved Neubauer China*, *Cat*. *No*. *1103*). For infection, 200 μl solution containing 5000 trypanosomes (*T*. *congolense*, *T*. *b*. *brucei*, *T*. *vivax* or *T*. *evansi*) was inoculated per mouse via intraperitoneal route. Mice were euthanized by CO_2_ gas at the first peak of parasitaemia (1x10^8^ trypanosomes/ml) and bled by cardiac puncture using a one ml syringe (*Kovax-syringe*, *Korea Vaccine Co*. *Ltd*) prefilled with heparin solution (40 μl). Obtained blood was pooled in a 15 ml centrifuge tube followed by centrifugation (1224 x *g*, 10 mins, 22°C). The buffy coat was collected and passed through a PD-10 column (*Cytiva*, *Cat*. *No*. *17043501*) packed with DEAE Sepharose Fast Flow matrix (*Cytiva*, *Cat*. *No*. *17070901*). The DEAE Sepharose Fast Flow matrix was pre-equilibrated with Phosphate Saline (PS) solution (NaCl, 36.5 mM; NaH_2_PO_4_, 3.6 mM; Na_2_HPO_4_, 59.5 mM) at either pH 7.5 (for *T*. *congolense* and *T*. *vivax*), or pH 8.0 (for *T*. *b*. *brucei* and *T*. *evansi*). Trypanosomes were eluted from the column using PS solution containing D-Glucose (88.8 mM) at either pH 7.5 or pH 8.0 for the respective species of trypanosomes. Eluted cells were centrifuged (1736 x *g*, 15 mins, 22°C) and obtained pellet was dissolved in 1xPBS (1000 μl). The lysate was prepared by resuspending the pellet followed by three rounds of freeze and thaw cycles alternating between -80°C and thaw 37°C, respectively. While on ice, the partially lysed cells were homogenized by sonication (*Ultrasonic Processor K-SuperSonic KSS-N900DT*, *Korea Process Technology Co*., *Ltd*.), and centrifuged (27,237 x *g*, 30 mins, 4°C). The supernatant was collected and the concentration of protein in the soluble lysate was estimated by a NanoDrop spectrophotometer and stored at—20°C.

Sera used in the experiment were harvested from uninfected (naïve) as well as trypanosome-infected mice. In brief, mice were bled into a 1.5 ml centrifuge tube and blood was stored at 4°C for 2 days followed by centrifugation (9425 x *g*, 10 mins, 4°C). Afterwards, sera were harvested by a micropipette and stored at -20°C.

### Recombinant proteins

All recombinant proteins used in this study originated from past studies. Nb474 fused with *his*_*x6*_ peptide tag (Nb474H), Nb474 fused with both *his*_*x6*_ and haemagglutinin (HA) peptide tags (Nb474HA), *Tco*ALD, *T*. *vivax* aldolase (*Tv*ALD), and *Leishmania mexicana* aldolase (*Lm*ALD) originated from [22]; *T*. *congolense* pyruvate kinase (*Tco*PYK) from [23]; and *T*. *evansi* enolase (*Tev*ENO) and Nb77 fused with *his*_*x6*_ peptide tag (Nb77H) from [27]. These proteins were produced, and purified by nickel affinity chromatography. The levels of production and purity of the recombinant proteins were analysed by SDS-PAGE. The concentration of the purified protein in the sample was measured by a NanoDrop and stored, in aliquots, at -20°C.

### Mice immunization and analysis of immune response

Prior to immunization, blood (2.5 μl) was collected by tail-snip from the BALB/c mice (*n* = 3), and it was diluted (1/200) in 1xPBS. The diluted blood samples were stored at -20°C until analysed by Ab-ELISA. On the day of immunization, *Tco*ALD was diluted to a desired concentration in a sterile distilled water and then emulsified in Gerbu Adjuvant (*Biotechnik GmbH*, *Cat*. *No*. *3001-1mL*) following the manufacturer’s guideline (Table 1). The Ag preparation was successively administered subcutaneously six times into scruff of the neck of the mice. Two days after the last booster shot, blood (2.5 μl) was collected from each of the immunized mice and it was diluted (1/200) in 1xPBS. The Ab levels in the blood sample preparations was analysed by ELISA.

**Table 1:** The immunization schedules per mouse.

Day	0	14	21	28	29	30
***Tco*ALD (μg)**	100	50	50	50	50	50
**Adjuvant (μl)**	40	20	20	20	-	-
**Distilled H**_**2**_ **O (μl)**	34	17	17	17	37	37
**Total dose (μl)**	100	50	50	50	50	50

### Construction of hybridoma library

A hybridoma library was generated using the *ClonaCell-HY Hybridoma* Kit (*STEMCELL Technologies*, *Cat*. *No*. *03800*). A mouse with the highest Ab response was euthanized on day eight post last booster shot, and the spleen was harvested in 5 mL *ClonaCell-HY Medium B (STEMCELL Technologies*, *Cat*. *No*. *03802*) followed by pulverisation in *Medium B* (5 ml) using a gentleMACS Dissociator (*Miltenyi Biotec*, *Cat*. *No*. *130-093-235*). The macerated cell suspension was sieved through a 70 μm strainer (*SPL cell strainer*, *Cat*. *No*. *93070*). The filtrate was diluted (1/6) in *Medium B* followed by centrifugation (316 x *g*, 10 mins, 22°C). This step was repeated twice. The washed cell pellet was resuspended in *Medium B* (25 ml) and cells were enumerated by a haemocytometer. Next, 1x10^8^ splenocytes were obtained for fusion with NS01 parental myeloma. For preparation of NS01 cells, the *Medium A* passaged cells (1x10^7^) were inoculated into a T-250 flask prefilled with *Medium A* (148 ml) a day before fusion. The cell suspension was later evenly distributed over 15 culture dishes (*SPL Life Sciences Co*., *Ltd*. *Cat*. *No*. *20100*) followed by an overnight incubation in a humidified CO_2_ incubator (5% CO_2_, 37°C). On the day of fusion, the cells were harvested by centrifugation (316 x *g*, 10 mins, 22°C) when they were in early-mid log phase (8.2x10^4^ cells/ml). The pellet was resuspended in *Medium B* (30 ml) followed by centrifugation (316 x *g*, 10 mins, 22°C). The washing step was repeated twice and the pellet was resuspended in *Medium B* (25 ml) followed by enumeration using a haemocytometer. For fusion, the splenocytes (9.5x10^7^cells in 19 ml) were mixed with the NS01 cells (1.6 x10^7^ cells in 25 ml) in a 50 ml centrifuge tube followed by centrifugation (316 x *g*, 10 mins, 22°C). The pellet was disrupted by gentle tapping. *ClonaCell-HY PEG* (1 ml) was added dropwise to the pellet by a one ml sterile transfer pipette (VWR, *Cat*. *No*. *VWRI612-1747)* over a period of one minute without stirring. The cells were resuspended by the tip of a serological pipette for one minute by a continuous gentle stirring. Then *Medium B* (4 ml) was dispensed, dropwise over a period of four minutes, into the fusion mixture while stirring in-between the additions until all the solution was ejected. Additional *Medium B* (10 ml) was slowly added to the fusion mixture followed by incubation (15 mins, 37°C) in a water bath. Afterwards, *Medium A* was added twice to the cells (starting with 30 ml and then 40 ml), and each of these additions was followed by centrifugation (316 x *g*, 7 mins, 22°C). The supernatant was carefully drained after the last wash leaving behind the pellet, which was then slowly resuspended in *ClonaCell-HY Medium C* (10 ml) and transferred into a T-75 cm^2^ cell culture flask (*CellStar cell culture flask Greiner bio one*, *Cat*. *No*. *658170*) prefilled with *Medium C* (20 ml). The cell mixture was incubated in a humidified incubator (5% CO_2_, 16 hrs, 37°C). The following day, fused cell suspension was transferred into a 50 ml centrifuge tube and centrifuged (316 x *g*,10 mins, 22°C). Obtained pellet was resuspended in *Medium C* (12 ml), and transferred into *ClonaCell-HY Medium D* (90 ml). The two solutions were mixed by gently inverting the bottle several times followed by incubation (5 mins, 37°C). The semi-solid medium was distributed (10 ml/plate) over ten 100 mm cell culture dishes. The seeded dishes were arranged in large square plates for a prolonged incubation. Each of the large square plates received at most three seeded dishes and a fourth dish filled with water only to provide local humidity. The assembled cultures were incubated for 10 days, without disturbance, in a humidified CO_2_ incubator (5% CO_2_, and 37°C).

### Hybridoma library screening and isotype characterization

The hybridoma library was screened for anti-*Tco*ALD mAb producing clones when the colonies were visible on the semi-solid medium eleven days after plating. During screening, a colony was drawn into a 10 μl micropipette tip and subsequently inoculated into a well of a 96-well cell culture (*SPL Life technologies*, *Cat*. *No*. *31096*) prefilled with *Medium E* (100 μl). When all the wells were seeded, additional *Medium E* (50 μl) was dispensed into each of the seeded wells followed by incubation in a humidified CO_2_ incubator (4 days, 37°C, 5% CO_2_). On day four, supernatant (50 μl) was harvested per mini-culture and probed by ELISA for the presence of mAbs against *Tco*ALD. For the ELISA, Half-Area ELISA plate (*Corning*, *Cat*. *No*. *3690*) was coated with *Tco*ALD (0.25 μg/well) diluted in 1xPBS for an overnight at 4°C. The wells were washed thrice with PBS-T. Blocking solution, 5% skimmed milk (*Oxoid*, *Cat No*: *LP00338*) in 1xPBS, was added (160 μl/well) to washed wells followed by incubation (2 hrs, 22°C). The blocking solution was discarded and the wells were washed thrice. Thereafter, the mini-culture supernatant (50 μl), previously harvested, was added into each of the wells followed by incubation (1 hr, 22°C). The supernatant was discarded and the wells were washed thrice. Goat anti-mouse immunoglobulin (Ig) horseradish peroxidase (HRP) (*SouthernBiotech SBA Clonotyping System-HRP*, *Cat*. *No*. *5300–05*) diluted (1/1000) in 2.5% milk solution was added into the wells (50 μl/well) followed by incubation (1 hr, 22°C). The unbound antibodies were washed four times. Thereafter, 3,3′,5,5′-Tetramethylbenzidine (TMB) substrate (*Sigma*, *T0440-100 ml*) was added (50 μl/well) followed by incubation (15 mins, 22°C). The reaction was stopped by adding 1M H_2_SO_4_ (50 μl/well).

The retrieved positive clones were subjected to a secondary screening to identify potential “false positive” clones producing mAbs against either *his*_*x6*_-tag on recombinant *Tco*ALD immunizing antigen, or *E*. *coli* expression host proteins, which co-purified with the recombinant *Tco*ALD. For this ELISA, all the positive clones were probed for binding unpurified recombinant *Tco*PYK and Nb474H crude protein extracts (both proteins are fused with *his*_*x6*_-tag and were not purified from the crude *E*. *coli* lysate), or purified Nb474H protein (a protein fused with *his*_*x6*_-tag), while the purified recombinant *Tco*ALD served as a positive control.

The clones that bound *Tco*ALD specifically were next (iso)typed (*SouthernBiotech SBA Clonotyping System-HRP*, *Cat*. *No*. *5300–05*). For (iso)typing, *Tco*ALD was coated (0.25 μg/well) followed by addition of supernatants (50 μl/well). Each of the supernatants was probed with assortment of goat anti-mouse HRP conjugates against mouse IgG1, IgG2a, IgG2b, IgG3 and IgM.

Finally, a third screening was done to verify if the characterized mAbs would detect *Tco*ALD, *Tco*PYK or 1xPBS in ELISA plate coated with Nb474H or Nb77H as capturing reagents.

### Production and purification of monoclonal antibody

The production of the only mAb clone (IgM8A2) that showed specific binding to *Tco*ALD was upscaled after adapting to *Hybridoma-Serum Free Medium* (SFM) (*gibco*, *Cat*. *No*. *12045–084*) and assessing its binding to *T*. *congolense* lysate (*Tco*Lys). During adaptation, the IgM8A2 expressing clone cultured in *Medium E* was progressively exposed to *Medium A*. For this, *Medium E* was gradually reduced, from the *Medium E-A* mixture, by 25% until attaining 0% while *Medium A* was increased by 25% until reaching 100%. At the final stage of adaptation to 100% *Medium A*, the culture (2 ml) was first pelleted by centrifugation (316 x *g*, 10 mins, 22°C) and then resuspended in 100% *Medium A* (3 ml). The cell suspension was dispensed into a well on cell culture plate (*SPL Life technologies*, *Cat*. *No*. *30006*) and incubated in a humidified CO_2_ incubator (37°C, 5% CO_2_). Afterwards, cells in *Medium A* were gradually adapted to SFM. As such, for complete adaptation to SFM, two cultures of 3 ml each, in a mixture of *Medium A* (25%) and SFM (75%) were pooled in a 15 ml centrifuge tube followed by centrifugation (316 x *g*, 10 mins, 22°C). The pellet was resuspended in SFM (6 ml) and the cell suspension was dispensed into two culture dishes (3 mL/dish) prefilled with SFM (12 ml/plate) followed by incubation in a humidified CO_2_ incubator (37°C, 5% CO_2_). At 1.74x10^6^/ml cell density, the culture (15 ml) was inoculated into a vented tissue flask-T 175 prefilled with SFM (30 ml) and incubated in a humidified incubator (37°C, 5% CO_2_). On day 4 post-inoculation when the culture has attained a stationary growth phase, characterized by yellowish coloration of the medium, the supernatant was harvested and centrifuged (316 x *g*, 10 mins, 22°C) followed by storage at -20°C. The purification of mAb was done by AKTA Start (*Cytiva*) employing *UNICORN start 1*.*1 (Build1*.*1*.*0*.*2)* software. During purification, 50 mL of the supernatant was defrosted and clarified by filtration through a 0.2 μM sieve (*Sartorius Minisart*, *S6534*). The filtrate was dialysed in 1M (NH_4_)_2_SO_4_, pH 7.5 (500 ml) at 4°C using a dialysis tubing (*Spectra/Por Dialysis Membrane Standard RC tubing*) of pore size 6-8kD. The dialysate (50 mL) was loaded (0.5ml/min) onto a HiTrap IgM purification column (*Cytiva*, *Cat*. *No*. *17-5110-01*), which was pre-equilibrated with five column volume of the *binding buffer* [20 mM Sodium Phosphate, 1M (NH_4_)_2_SO_4_, pH 7.5]. Thereafter, copious quantity of *binding buffer* was passed through the column to wash-off the unbound impurities until A_280_ returned to baseline. The retained Ab was eluted out of the column by washing with *elution buffer* (20 mM Sodium Phosphate Buffer, pH 7.5) at 0.5 ml/min. The eluted Ab was dialysed in 1xPBS pH 7.4 at 4°C. The concentration of Ab in the sample was estimated by a NanoDrop spectrophotometer and the sample was stored at -20°C.

### Biotin-labelling of monoclonal antibody

The IgM8A2 monoclonal antibody was labelled with biotin, using the *EZ-Link Sulfo-NHS-Biotin* kit (*Thermo Scientific*, *Cat*. *No*. *21217*). Biotin powder (4 mg) in the kit was reconstituted in distilled water (500 μl) and the solution (40 μl) was added into a IgM8A2 solution (1000 μl), which was at a concentration of 1 mg/ml. The mixture was incubated on ice for an overnight. Thereafter, the biotin-labelled IgM8A2 (IgM8A2-B) was dialysed in 1xPBS pH 7.4 using *Slide-A-Lyzer 3*.*5 K Dialysis Cassettes* (*Thermo Scientific*, *Cat*. *No*. *66330*). The concentration of IgM8A2-B in the solution was measured by a NanoDrop spectrophotometer and the sample was stored at -20°C. The success of IgM8A2 biotin-labelling was ascertained by ELISA employing the HRP Streptavidin (Strep-HRP) (*Biolegend*, *Cat*. *No*. *405210 / 1 ml*) and TMB reporter system.

### Assessing binding properties of retrieved monoclonal antibody

Binding of IgM8A2 to a denatured aldolase was assessed by western blot and an indirect ELISA. For western blot, aldolase was resolved by SDS-PAGE under denaturing condition and electroblotted onto nitrocellulose membrane (*Thermo scientific*, *Cat*. *No*. *88018*). The blotted protein was probed with a solution of an unlabelled IgM8A2 (4.47 μg/ml) in 2.5% milk followed by goat anti-mouse IgM HRP (*SouthernBiotech*, *Cat*. *No*. *5300–05*) diluted (1/1000) in 2.5% milk. The retention of conjugated anti-mouse IgM by IgM8A2 was revealed by incubation in HRP substrate solution [10 ml 99% methanol (10 ml), 4-Chloro-1-napthol powder (45 mg), 1xPBS (45 ml), and 30% hydrogen peroxide (100 μl)]. An indirect ELISA was used to investigate the binding of IgM8A2 to a heat-denatured aldolase. Briefly, recombinant *Tco*ALD or *Tv*ALD was diluted to 200 μg/ml, and aliquoted (120 μl/vial) followed by incubation at 55°C for different time lengths (0, 10, 20, 30, 40, 50, and 60 mins). The samples were coated (10 μg/well) on ELISA plate for an overnight at 4°C. The coating was discarded and wells were blocked with 5% milk solution (160 μl/well) for 2 hrs at 22°C. The wells were washed thrice and IgM8A2 diluted to 4.74 μg/ml in blocking buffer was added (50 μl/well) followed by incubation (1 hr, 22°C). Wells were washed 4 times and a dilution (1/1000) of goat anti-mouse IgM HRP was added (50 μl/ well) followed by incubation (1 hr, 22°C). Wells were washed-off excess unbound HRP conjugate five times. TMB substrate (*Sigma*, *T0440-100 ml*) was added (50 μl/well) and incubation was allowed for 15 min. The reaction was stopped with 1M H_2_SO_4_ (50 μl/well) and OD_450nm_ was read.

Next, the binding of IgM8A2 to the aldolases of other livestock infective trypanosome species, besides *T*. *congolense*, was assessed by an indirect immunofluorescent assay. Fixed-permeabilized trypanosomes were incubated in IgM8A2-B solution (2.5 μg/ml) or Biotin anti-mouse IgM Antibody (*Biolegend*, *Cat*. *No*. *406504/500 μg*) solution (2.5 μg/ml) followed by Cy3 Streptavidin (*Biolegend*, *Cat*. *No*. *405215*) solution (1 μg/ml) as described (S1 Materials and Methods).

The specificity of Nb474H/IgM8A2-B “hybrid” sandwich system for detection of aldolase was assessed on recombinant *Tco*ALD, *Tev*ENO or 1xPBS by ELISA employing the Strep-HRP and TMB reporter system.

Finally, to asses the competition for binding *Tco*ALD between IgM8A2 (Ag-detection reagent) and Nb474 (Ag-capture reagent), a competition ELISA was conducted as described (S2 Materials and Methods).

### ELISA titration of antigen capture Nanobody against detection monoclonal antibody

Nb474H (5 μg/ml) was serially diluted (two-fold) in 1x PBS until a final concentration of 0.005 μg/mL. Except for the wells on column 12, which were filled with 1xPBS (50 μl/well), the dilutions were coated (50 μl/well) on respective wells (S1 Table) followed by overnight incubation at 4°C. Next, wells were emptied, washed thrice, and *SuperBlock* blocking buffers (*thermoscientific*, *Cat*. *No*. *37516*) was added in coated wells (160 μl/well) for 2 hrs at 22°C with an hourly buffer refreshment. Afterward, the blocking buffer was emptied and the wells were washed thrice. A constant amount of *Tco*ALD antigen was added across the plate (0.25 μg/well) followed by incubation for an hour at 22°C. Wells were washed thrice. Next, two-fold serial dilutions of IgM8A2-B, starting from 5 μg/mL until 0.08 μg/ml, were added row-wise in a decreasing concentration except for wells in row H, which received 1xPBS only (S2 Table). The reaction was incubated for an hour at 22°C and the wells were washed four times. Strep-HRP diluted to 0.5 μg/ml in *SuperBlock* was added (0.025 μg/well) into the wells followed by incubation for an hour at 22°C. The enzyme conjugate was emptied and the wells were washed five times. TMB substrate (*Sigma*, *T0440-100 ml*) was added (50 μl/well) and color development was allowed for 15 mins. The reaction was stopped by adding 1M H_2_SO_4_ (50 μl/well) and OD_450nm_ was read.

### Analytical sensitivity of N474H/IgM8A-B “hybrid” sandwich ELISA

A fixed amount of Nb474H was coated (0.1 μg/well) in wells on rows *B-E*, columns *2–11* and incubated for an overnight at 4°C. The following morning, coating solution was emptied and the wells were washed thrice. Washed wells were blocked by adding *SuperBlock* (160 μl/well) for 2 hrs at 22°C with an hourly refreshment. The blocking solution was discarded and wells were washed thrice. Serial dilutions (two-fold) of *Tco*ALD (100–0.0004 μg/ml) were added into duplicate wells with control wells (D11E11) receiving 1xPBS only (S3 Table). The reaction was incubated for an hour at 22°C. The unbound antigens were discarded and the wells were washed thrice. The IgM8A2-B diluted to 2.5 μg/mL in *SuperBlock* was added into the wells (50 μl/well) followed by incubation for an hour at 22°C. Wells were washed four times and Strep-HRP diluted to 0.5 μg/ml in blocking buffer was added (50 μl/well) followed by an hour of incubation at 22°C. The HRP conjugate was discarded and wells were washed five times. TMB substrate (*Sigma*, *T0440-100 ml*) was added (50 μl/well) into the wells and incubated for 15 mins. The reaction was stopped by adding an equal volume of 1M H_2_SO_4_ solution and the absorbance was read at 450nm.

### Detection of *T*. *congolense* infections by Nb474H/IgM8A2-B “hybrid” sandwich ELISA

Sera collected from the naïve or mice experimentally infected with a panel of trypanosome species including *T*. *congolense* TC13 at parasitemia 1x10^8^ trypanosomes/ml, *T*. *b*. *brucei* AnTat1.1E at parasitemia 4.8x10^8^ trypanosomes/ml, *T*. *evansi* STIB 816 at parasitemia 2.7x10^8^ trypanosomes/ml, or *T*. *vivax* ILRAD 700 at parasitemia 4.1x10^8^ trypanosomes/ml were examined with the Nb474H/IgM8A2-B “hybrid” sandwich ELISA. Nb474H was coated (0.1 μg/well) and incubated for an overnight at 4°C. Wells were emptied and washed thrice. Washed wells were blocked with 10% BSA (*BOVOGEN*, *Cat*. *No*. *BSAS 0*.*1*) at 160 μl/well and incubated for an hour at 22°C. The blocking buffer was refreshed and incubation was continued for an hour before discarding. Undiluted sera were added (50 μl/well) in duplicate into emptied wells. Both recombinant *Tco*ALD (10 μg/well) and *T*. *congolense* lysate (5 μg/well) were used as positive controls for the ELISA. Incubation was performed for one hour at 22°C. The unbound antigens were discarded and the wells were washed thrice. Next, IgM8A2-B diluted to 2.5 μg/ml in 10%BSA was added into the wells (50 μl/well) followed by incubation for an hour at 22°C. The wells were washed four times. A strep-HRP solution diluted to 0.25 μg/ml in 10% BSA was added into the wells (50 μl/well) followed by incubation for 30 mins at 22°C. The conjugate was discarded and the wells were washed six times. Thereafter, color development was initiated by adding TMB substrate (*Sigma*, *T0440-100 ml*) at 50 μl/well. Incubation was allowed for 15 mins and the reaction was stopped by adding 1M H_2_SO_4_ (50 μl/well) followed by OD reading at 450nm.

### Binding of native *T*. *congolense* aldolase by Nb474H/IgM8A2-G sandwich in a dot blot system

The potential for translation of Nb474 and Gold-labelled IgMA82 (IgM8A2-G) “hybrid” sandwich technology into a LFA device was demonstrated in a dot-blot assay. The IgM8A2 was labelled with Gold 20nm using a Gold Conjugation Kit (*abcam*, *Cat*. *No*. *ab188215*) according to the manufacturer’s protocol. Specifically, IgM8A2 was dialysed in 2-(N-Morpholino) ethanesulfonic acid hydrate, 4-Morpholineethanesulfonic acid (MES hydrate) buffer at pH 7.4. The dialysed mAb was later diluted to 0.25 mg/ml in an *Ab diluent* provided by the kit. Thereafter, an aliquot (12 μl) of the diluted mAb was mixed with the kit’s *Reaction Buffer* (42 μl). The mixture (45 μl) was dispensed into a vial of lyophilized Gold 20 nm followed by reconstitution and then incubation (15 mins, 22°C). A *Quencher* solution, provided by the kit, was added (5μl) into the mixture. To wash off unlabelled mAb, a solution of the *Quencher* diluted (1/10) in distilled water was added to the mixture followed by centrifugation (9000 x *g*, 22°C). The supernatant was carefully drawn and the resultant pellet was resuspended in a fresh diluted *Quencher* (50 μl). Thereafter, the binding of IgM8A2-G to test proteins including *Tco*ALD, *T*. *congolense* lysate (*Tco*Lys), *Lm*ALD, and *Tev*ENO was examined. The test proteins were either blotted direct on a nitrocellulose membrane (S4 Table) or anchored onto a nitrocellulose membrane by a blotted Nb474H capture reagent (S5 Table). To examine IgM8A2-G binding to test or control proteins blotted direct on the nitrocellulose membrane, each of the test samples was spotted (30 μg/spot) into a “well” encircled by an adhesive label (*Chengu 400 Pieces Self-Adhesive Reinforcement Labels*). When the sample spots were damp-dried, the membrane was blocked by immersing into a 5% milk solution and incubated with gentle rocking for an overnight at 22°C. The blocking buffer was rinsed thrice with PBS-T and IgM8A2-G solution was spotted (10 μl/spot) on the “wells” previously blotted with the samples. The reaction was allowed to proceed for 45 mins at 22°C. The membrane was washed thrice with PBS-T with each wash lasting for 30 mins. The membrane was blot-dried and photographed. To examine IgM8A2-G binding to proteins captured by blotted Nb474H, the Nb474H was spotted on the membrane (150 μg/spot) and allowed to damp-dry. Afterwards, the membrane was blocked with 5% milk for an overnight at 22°C. The membrane was rinsed thrice. Thereafter, each of the test samples was separately spotted (90 μg/spot) in one of the “wells” previously spotted with Nb474 followed by incubation (45 mins, 22°C). The membrane was washed thrice with PBS-T and each of the washings lasted 30 mins. Afterward, the membrane was blot-dried and the photograph was acquired by a camera.

## Results

### Screening hybridoma library retrieved a specific anti-trypanosome aldolase monoclonal antibody

**Fig 1:**
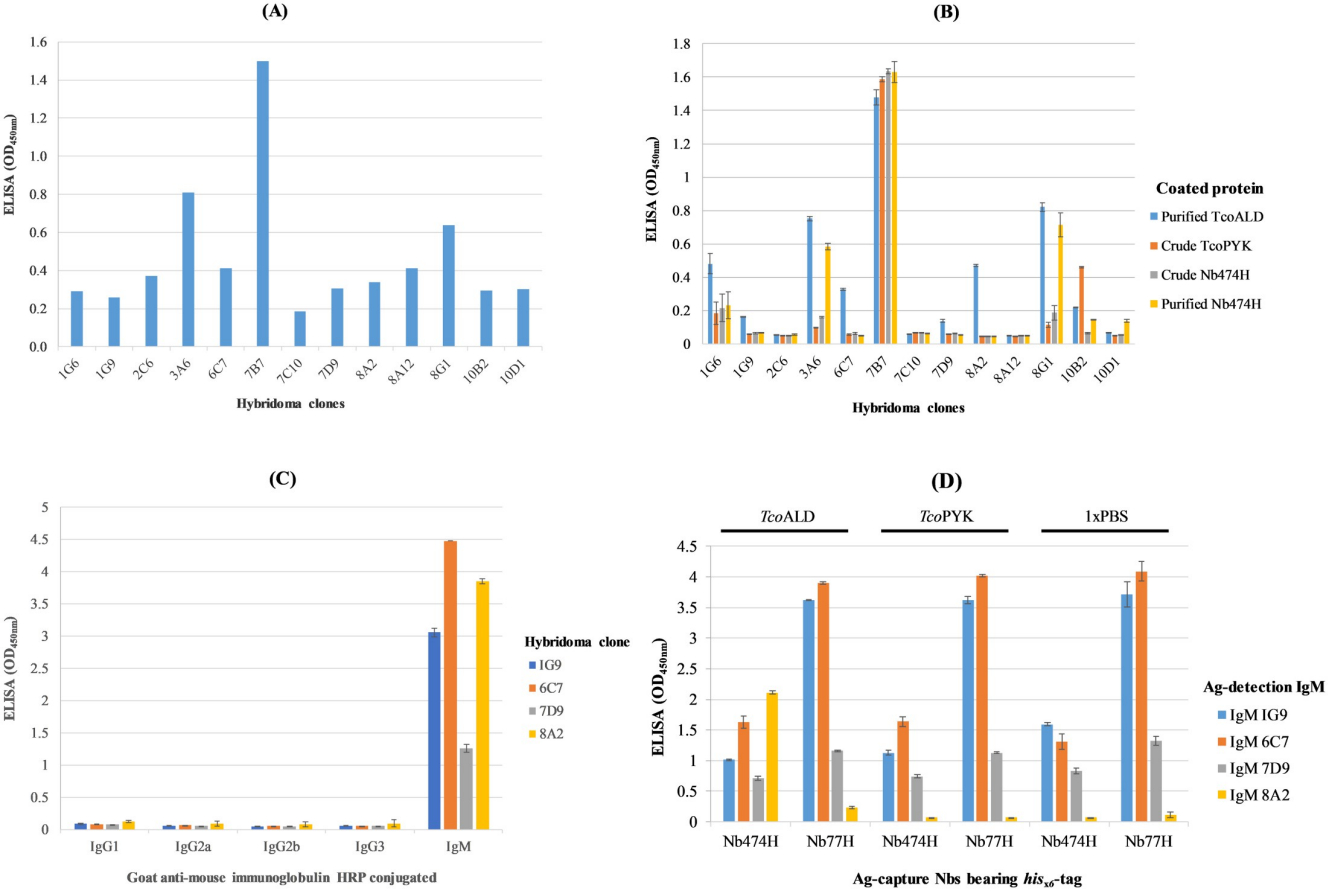
Selection of clones producing anti-*Tco*ALD monoclonal antibody (mAb) from hybridoma library. (A) Primary screening of the hybridoma library identified 13 positive hybridoma clones. Threshold OD score for positive clones was ≥ 0.2 units. (B) Positive hybridoma clones subjected to a secondary screening on *Tco*ALD or irrelevant proteins. Clones 1G9, 6C7, 7D9 and 8A2 produced *Tco*ALD specific binders; clones 1G6, 3A6, 7B7, 8G1, and 10B2 produced binders of both *Tco*ALD and irrelevant protein; and each of the clones 2C6, 7C10, 8A12, and 10D1 has no *Tco*ALD binder. (C) Isotyping mAbs showing specific binding to *Tco*ALD. Going by their reactivity with the goat anti-mouse immunoglobulin M antibody isotype, all the mAbs belonged to the IgM class. (D) IgM8A2 detects *Tco*ALD in a sandwich combination with Nb474H. IgM clones were analysed for specific detection of *Tco*ALD in a sandwich combination with Nb474H (anti-*Tco*ALD Nb) or irrelevant Nb77H (anti-*Tev*ENO Nb). Only IgM8A2 specifically bound *Tco*ALD in a sandwich combination with Nb474H.

To induce B cell clones expressing anti-*Tco*ALD specific antibodies for construction of hybridoma library, mice were immunized with recombinant *Tco*ALD. Comparing ELISA signals (OD_450nm_) on blood samples collected before (pre-immune) and after immunization (immune), it was conceivable that immunization of mice with *Tco*ALD evoked Ab response (S1 Fig). Afterwards, hybridoma library was constructed from B-cells of a mouse with the highest Ab response and the library was screened on *Tco*ALD. A primary screening of the hybridoma library retrieved 13 reactive clones. A clone was considered reactive when the OD_450nm_ was ≥ 0.2 units (Fig 1A). Of the 13 clones, two (1G6 and 1G9) originated from *plate 1*, one (2C6) from *plate 2*, one (3A6) from *plate 3*, one (6C7) from *plate 6*, three (7B7, 7D9, and 7C10) from *plate 7*, three (8A2, 8A12, and 8G1) from *plate 8*, and two (10B2 and 10D1) from *plate 10*. The consistency and specificity of the 13 reactive clones for binding *Tco*ALD was again verified by a secondary screening on *Tco*ALD or irrelevant proteins including *his*_*x6*_-tag bearing *Tco*PYK in *E*. *coli* BL21 (DE3) crude lysate, *his*_*x6*_-ta*g* bearing Nb474 (Nb474H) in *E*. *coli* WK6 lysate, and purified Nb474H. Four of the clones (1G9, 6C7, 7D9 and 8A2) showed unique *Tco*ALD specificity, while five clones (1G6, 3A6, 7B7, 8G1, and 10B2) produced antibodies that bound both *Tco*ALD as well as irrelevant proteins. In addition, four clones (2C6, 7C10, 8A12, and 10D1), which had scored positive in the primary screening turned out negative in the secondary screening (Fig 1B). To check Ab isotype expressed by each of the four hybridoma clones (1G9, 6C7, 7D9 and 8A2), the clones were probed with goat anti-mouse HRP conjugates against mouse IgG1, IgG2a, IgG2b, IgG3 or IgM. Surprisingly, all the four hybridoma clones 1G9, 6C7, 7D9 and 8A2 were IgM as inferred from specific reactivity with goat anti-mouse IgM HRP (Fig 1C). Next, each of the four IgM producing clones was verified for reactivity with *Tco*ALD, *Tco*PYK, or PBS added into the ELISA wells precoated with *hisx6*-tag Nb474 (Nb474H) or *his*_*x6*_-tag Nb77 (Nb77H) capturing reagent. Unlike the three IgM clones (1G9, 6C7, and 7D9) that showed indiscriminate reactivity, IgM clone 8A2 reacted with *Tco*ALD dispensed into wells precoated with Nb474H only (Fig 1D).

### Binding characteristics of the anti-trypanosome aldolase monoclonal antibody

**Fig 2:**
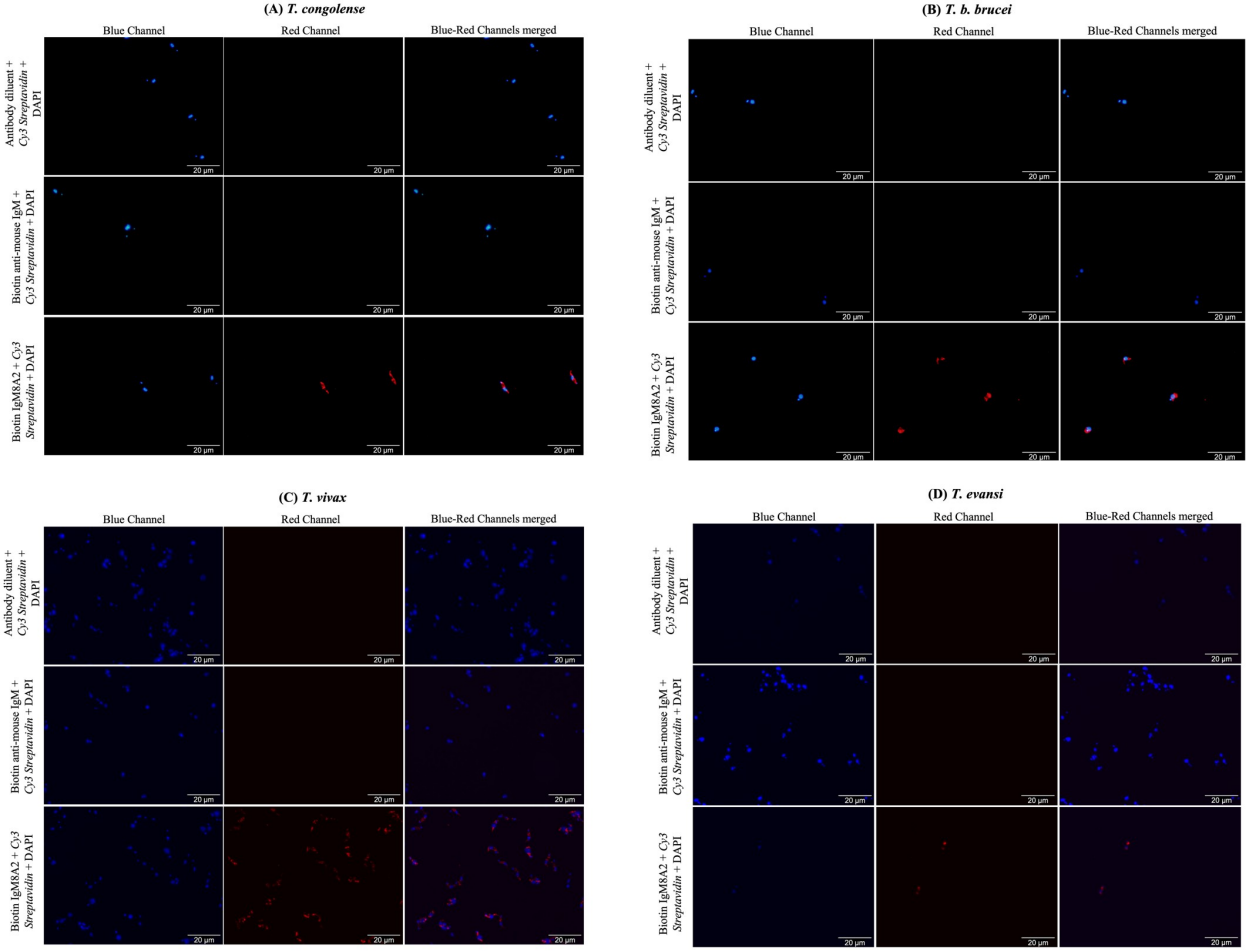
*In situ* indirect labelling of trypanosome glycosome with a biotinylated IgM8A2 (IgM8A2-B) primary antibody. Fixed and permeabilized *T*. *congolense* (A), *T*. *b*. *brucei* (B), *T*. *vivax* (C) or *T*. *evansi* (D) was probed in succesion with IgM8A2-B, biotin anti-mouse IgM or diluent only followed by *Cy3 streptavidin* red fluorescein conjugate. Red staining of trypansomes was observed across all the species where IgM8A2-B followed by *Cy3 streptavidin* red fluorescein conjugate was added pointing to cross-reactivity of the IgM8A2.

It was necessary to know whether IgM8A2 mAb also binds a denatured aldolase target. This is an attribute required to inform specimen handling and processing prior to testing. Western blot conducted on *Tco*ALD resolved under denaturing conditions did not show binding of the IgM8A2 to a nitrocellulose membrane-blotted *Tco*ALD protein. When binding of IgM8A2 to a heat-denatured recombinant *Tco*ALD or *Tv*ALD was also assessed by ELISA, only baseline signals characterised heat-treated samples (S2 Fig). These two experiments indicate denaturation of *Tco*ALD abolish recognition by IgM8A2. Secondly, cross-recognition of native aldolases from other important species of livestock trypanosomes including *T*. *congolense*, *T*. *b*. *brucei*, *T*. *vivax* and *T*. *evansi* by IgM8A2 was analysed by an indirect immunofluorescence assay. Cross-reactivity of IgM8A2 *Tco*ALD Ag-detection reagent may find application in the development of a trypanosome pan-reactive multiplex LFA comprised of multiple trypanosome species specific aldolase Ag-capture reagents and a pan-reactive IgM8A2 trypanosome aldolase Ag-detection reagent. Therefore, cross-recognition of native aldolases from different trypanosomes was assessed *in situ* by incubating fixed and permeabilized trypanosome cells in a solution of biotin-labelled IgM8A2 (IgM8A2-B), and the binding of IgM8A2 was probed with *Cy3 strepatavidin* red fluorescein conjugate. It was discovered that IgM8A2 binds fixed and permeabilized trypanosome irrespective of the species *T*. *congolense*, *T*. *b*. *brucei*, *T*. *vivax* or *T*. *evansi*. (Fig 2A–2D).

**Fig 3:**
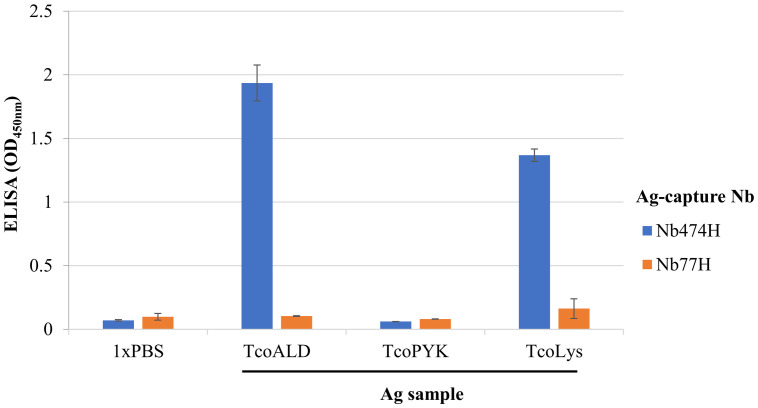
Detection of native aldolase in the lysate of *T*. *congolense* (*Tco*Lys) by Nb474H/IgM8A2 “hybrid” sandwich ELISA. Test samples including recombinant *T*. *congolense* aldolase (*Tco*ALD), *T*. *congolense* pyruvate kinase (*Tco*PYK) and *Tco*Ly added to wells pre-coated with Nb474H or Nb77H was each probed with IgM8A2. High signal intensity was observed in wells pre-coated with Nb474H capture reagent followed by addition of *Tco*ALD or *Tco*Lys, and then IgM8A2. From this finding it is plausible to conclude that Nb474H/IgM8A2 “hybrid” sandwich ELISA binds recombinant as well as native *Tco*ALD.

Thirdly, it was necessary to authenticate that IgM8A2, generated against recombinant *Tco*ALD, also binds native *Tco*ALD in a sandwich combination with Nb474H. This information would pave way for further progression with the development of the immunoassay meant for detection of native *Tco*ALD released in blood of animals with ongoing *T*. *congolense* infections. For this experiment, a *T*. *congolense* lysate (*Tco*Lys) preparation was used as a source of native *Tco*ALD. We demonstrated that IgM8A2 binds native *Tco*ALD, selectively captured by Nb474H, from a repertoire of proteins in the *Tco*Lys. This evidence was adduced from a high signal intensity (OD_450nm_) observed in the *Tco*ALD-treated well, which was comparable to *Tco*Lys-treated (Fig 3).

Fourthly, we investigated if Nb474 (*Tco*ALD Ag-capture reagent) and IgM8A2 (*Tco*ALD Ag-detection reagent) bind a common epitope. In the event that both Nb474 and IgM8A2 bind a common epitope on *Tco*ALD, translation of the immunoassay from an ELISA to a LFA would be technically impossible. A prior exposure of *Tco*ALD to IgM8A2 Ag-detection reagent, as it is the case with LFA, would lead to epitope masking by IgM8A2 such that only a few or even no will be left on *Tco*ALD for the binding of Nb474 Ag-capture reagent. The likelihood of common epitope binding by the two reagents was assessed by a competitive ELISA. The ELISA showed that Nb474 and IgM8A2 do not interfere with each other’s binding to *Tco*ALD. Premixing of Nb474HA (serving as a *Tco*ALD detection reagent) with a varying concentration (high to low) of IgM8A2 (serving as a competing reagent) followed by addition of the mixture (Nb474HA and IgM8A2) to *Tco*ALD did not interfere with the binding of Nb474HA to *Tco*ALD, and *vice versa*. In both ELISA, a constant high signal (OD_450nm_) was recorded across all the different concentration of the competing reagents (S3A and S3B Fig).

### A Nb474H/IgM8A2-B “hybrid” sandwich ELISA was developed and evaluated for detection of aldolase

**Fig 4:**
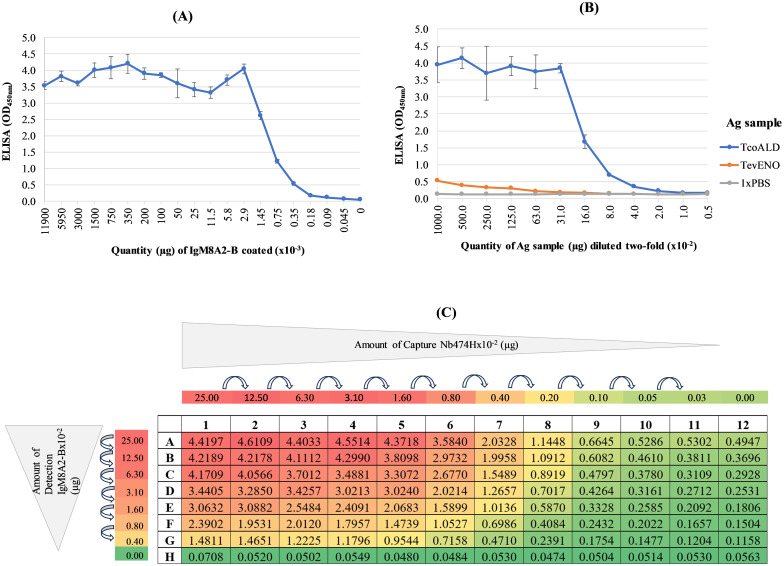
Development of the Nb474H/IgM8A2-B “hybrid” sandwich ELISA prototype. (A) IgM8A2 was labelled with biotin (IgM8A2-B) and validated by ELISA employing Strep-HRP TMB substrate reporter system. A high signal intensity (OD_450nm_) observed at higher concentrations of IgM8A2-B tapered with the decreasing concentrations of the mAb indicating that Strep-HRP was binding coated IgM8A2-B. (B) Nb474H/IgM8A2-B “hybrid” sandwich ELISA prototype binds *Tco*ALD. Compared to *Tev*ENO-treated wells where signal intensity remained baseline throughout different concentrations of the protein, a higher signal intensity proportional to the concentrations of *Tco*ALD was observed indicating that the prototype ELISA was binding *Tco*ALD. (C) Nb474H titrated against IgM8A2-B to find effective matching working concentration. The signal intensity was proportional to the amount of Nb474H and IgM8A2-B reagents used. High OD_450nm_ readings were consistent with wells treated with high concentrations of the Nb or IgM and vice versa. A standard working concentration of both Nb474H and IgM8A2-B was deduced to be 0.25 μg/ml per 50 μl because it was the least concentration of reagents, which was still giving a relatively high signal.

A Nb474H/IgM8A2-B “hybrid” sandwich ELISA was developed, and the working concentration of each of the Ag binding reagents, Nb474H Ag-capturing reagent and biotin-labelled IgM8A2 (IgM8A2-B) Ag-detection reagent, was optimized on *Tco*ALD prior to evaluation of the “hybrid” immunoassay for detection of trypanosome infections in mice. As a first step, recognition of IgM8A2-B Ag-detection reagent by Strep-HRP was assessed by ELISA. Signal intensity (OD_450nm_) observed on probing a coated IgM8A2-B with Strep-HRP and TMB substrate reporter system was proportional to the concentration of the coated biotin-labelled mAb (Fig 4A) signifying that, indeed, Strep HRP was binding the coated biotin-conjugate. Next, the detection of *Tco*ALD by Nb474H/IgM8A2-B “hybrid” sandwich ELISA was assessed against *Tev*ENO or 1xPBS controls. Higher signal intensity (OD_450nm_) was recorded in wells treated with *Tco*ALD sample than in the control wells treated with *Tev*ENO or 1xPBS (Fig 4B) proving that Nb474H/IgM8A2-B “hybrid” sandwich ELISA ably detects *Tco*ALD. Subsequently, the working concentration of each of the Nb474H and IgM8A2-B test reagents required for detection of *Tco*ALD was determined by a checkerboard titration. Qualitative (heatmap) as well as quantitative (OD_450nm_) data obtained from the titration experiment suggests the working concentration for each of the Nb474H and IgM8A2-B reagents falls about 0.25 μg/ml per 50 μl diluent (Fig 4C). Thereafter, the analytical detection limit of the optimized “hybrid” sandwich ELISA was also determined by titration on an in-house recombinant *Tco*ALD. The least amount of *Tco*ALD, which could be detected by the assay was 78.13 ng. This quantity of *Tco*ALD was the least that gave a signal intensity (OD_450nm_) two-fold above the negative control (S4 Fig).

### A Nb474H/IgM8A2-B “hybrid” sandwich ELISA detects active *T*. *congolense* infections

**Fig 5:**
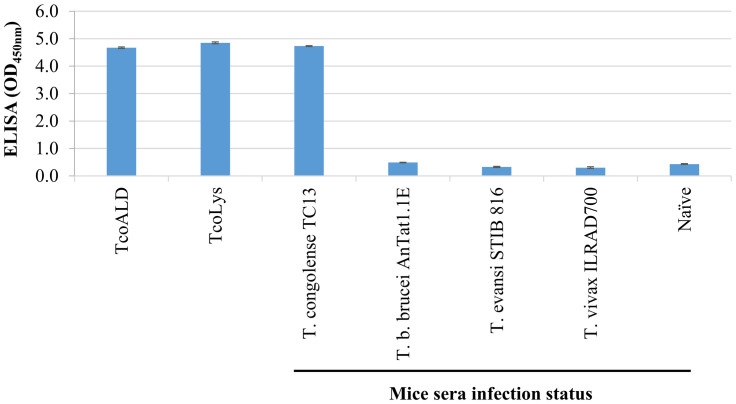
Nb474H/IgM8A2-B “hybrid” sandwich ELISA specifically detects *T*. *congolense* infection in mice. A panel of specimens including recombinant *T*. *congolense* aldolase (*Tco*ALD), *T*. *congolense* lysate (*Tco*Lys), and sera collected from trypanosome infected or naïve mice were probed with Nb474H/IgM8A2 sandwich ELISA. High signal intensity, comparable to *Tco*ALD or *Tco*Lys, was recorded in wells treated with sera collected from *T*. *congolense* TC13 infected mice (1x10^8^ trypanosomes/ml) indicating capability of the ELISA for detection of *T*. *congolense* infections. No reaction was observed in wells treated with naïve or sera collected from mice infected with *T*. *b*. *brucei* AnTat1.1E (4.8x10^8^ trypanosomes/ml), *T*. *evansi* STIB 816 (2.7x10^8^ trypanosomes/ml) or *T*. *vivax* ILRAD700 (4.1x10^8^ trypanosomes/ml).

A proof-of-concept detection of native *Tco*ALD circulating in sera of mice infected with *T*. *congolense* by the optimized Nb474H/IgM8A2-B “hybrid” sandwich ELISA was demonstrated to establish potential application of the assay for detection of *T*. *conglense* infections. A panel of mice sera collected from naïve animals or trypanosome infected (*T*. *congolense* TC13, parasitaemia 1x10^8^ trypanosomes/ml; *T*. *b*. *brucei* AnTat1.1E, parasitaemia 4.8x10^8^ trypanosomes/ml; *T*. *evansi* STIB 816, parasitaemia 2.7x10^8^ trypanosomes/ml; or *T*. *vivax* ILRAD700, parasitaemia 4.1x10^8^ trypanosomes/ml) was probed with Nb474H/IgM8A2-B “hybrid” sandwich ELISA. Signal intensity (OD_450nm_) recorded from wells treated with *T*. *congolense* infected mice sera was 10-fold higher than those wells treated with naïve mice sera or sera collected from mice infected with other trypanosome species (Fig 5) suggesting capability of the “hybrid” sandwich ELISA for specific detection of *T*. *congolense* infected animals.

### Gold-labelled IgM8A2 binds native *Tco*ALD in a dot blot assay

**Fig 6:**
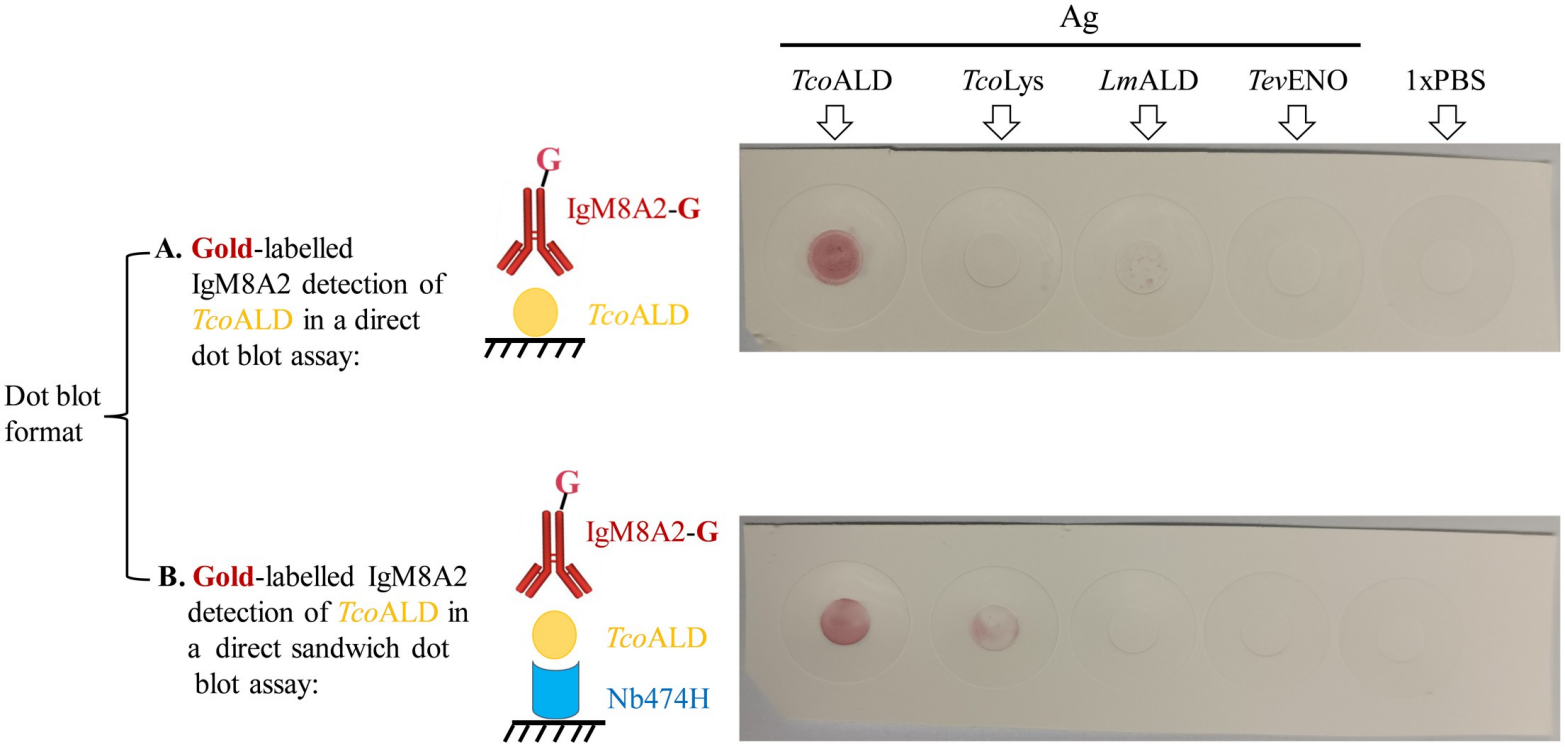
Dot blot detection of aldolase using Gold-labelled IgM8A2 (IgM8A2-G). (A) Binding of IgM8A2-G to antigen (Ag) including recombinant *T*. *congolense* aldolase (*Tco*ALD), *T*. *congolense* lysate (*Tco*Lys), recombinant *L*. *mexicana* aldolase (*Lm*ALD), recombinant *T*. *evansi* enolase (*Tev*ENO) or 1xPBS directly blotted on the nitrocellulose membrane. IgM8A2-G specifically labelled the spot blotted with *Tco*ALD. (B) A sandwich setup showing the binding of IgM8A2-G to Ag captured by Nb474H. Signal was observed in wells spotted with the *Tco*ALD and *Tco*Lys indicating the ability of IgM8A2-G for binding both recombinant as well as native aldolase.

To assess if, indeed, an opportunity exists for translation of the Nb/mAb “hybrid” sandwich ELISA prototype into a LFA, a dot blot assay employing *Au*-labelled IgM8A2 (IgM8A2-G) as an Ag-detection reagent was performed. First, the binding of IgM8A2-G to a recombinant *Tco*ALD or its native form in *T*. *congolense* lysate (*Tco*Lys) was assessed on proteins that were directly blotted on a nitrocellulose membrane. Next, the detection by IgM8A2-G of recombinant *Tco*ALD or its native form in *Tco*Lys was assessed on proteins anchored onto the nitrocellulose membrane by a spotted Nb474H Ag-capture reagent. Staining of nitrocellulose membrane occurred on the spot directly blotted with recombinant *Tco*ALD only (Fig 6A). On the second membrane where recombinant *Tco*ALD or its native form in *Tco*Lys samples was anchored by Nb474H, staining occurred on spots treated with recombinant *Tco*ALD and *Tco*Lys (Fig 6B). These results show that IgM8A2-G, in a sandwich combination with Nb474H, binds recombinant as well as native *Tco*ALD.

## Discussion

The Pan African Tsetse and Trypanosomiasis Eradication Campaign (PATTEC) coordinates the implementation of activities aimed at eliminating the tsetse fly vector and trypanosomiasis from the African continent [29]. A key factor undermining the eradication campaign is the inadequacies of current diagnostics for trypanosomiasis, which may make achievement of the set target unrealistic, in particular when it comes to the animal disease variants of trypanosomiasis. Undoubtedly, not many livestock farms have integrated trypanosomiasis diagnosis in their system. The deployment of ideal POCT for AAT in endemic areas for routine diagnosis, would change the trajectory. Unfortunately, such a test is yet non-existent. For this reason, clinicians often rely on clinical signs to arrive at a tentative diagnosis. Diagnosis of trypanosomiasis relying on clinical signs only, is grossly imperfect because the disease does not have pathognomonic signs for discrimination from other diseases that present similar signs. The elimination campaign would succeed faster with the deployment of effective POCT for active screening, monitor therapeutic efficacy, and disease surveillance.

Abs have been used for the development of several commercial Ag detection POCT devices for infectious diseases [30]; however, no such commercial Ag detection test exists for AAT [31] despite decades of active research. Hope for realization of the Ag detection tests for AAT has been raised with the invention of Nb technology, as a potential substitute for the conventional Abs. Although it has numerous desirable attributes [32,33], Nbs have not been completely free of hurdles [33]. Related to diagnostic applications, conjugation of Nbs to high molecular weight reporter molecules, including nanoparticles, enzymes and biotin for employment as Ag detection reagents in immunoassay, often presents a challenge. However, we have now shown herein that this challenge could be side-stepped by incorporation of a mAb as a detection reagent, ending up with a Nb/mAb “hybrid” Ag detection test device for trypanosomiasis. Specifically, a prototype antigen detection ELISA unit for detection of *T*. *congolense* infections was derived from a Nb (Nb474) and a mAb (IgM8A2) heterologous reagents targeting aldolase of the parasite. While Nb474H originated from an alpaca, IgM8A2 is of mouse origin.

A three-stage screening of the hybridoma library recovered an anti-*Tco*ALD specific IgM (IgM8A2). The primary screening recovered 13 positive clones, re-screening of the 13 positive clones recovered four clones, and a final re-screening of these four clones, with Nb474H employed as an antigen capture reagent, revealed IgM8A2 as a *Tco*ALD specific binder. The multi-stage screening employed identified among the positive clone non-specific binders, which were subsequently discontinued from downstream experiments. Although no further investigation was carried to ascertain the precise targets of these non-specific binders, binding to *his*_*x6*_-tag was postulated, given their consistent reaction with recombinant *his*_*x6*_-tag proteins. Noted were a few clones that scored positive in the primary screening and negative in the secondary screening. False positive reactions, characterized by high background signals, offer explanation for the observed status change. The fact that only a single anti-*Tco*ALD mAb producing clone could be retrieved from a large collection, suggests the immunized mouse evoked a low immune response to the Ag. Aldolase is a glycolytic enzyme of eukaryotes [34] meaning that *Tco*ALD partly qualifies as a self-Ag in a mouse model, potentially explaining its poor immunogenicity [35]. It is suggested that the developers of trypanosome Ag detection tests could pursue targeting high copy number low immunogenic Ags, as a way of overcoming low sensitivity resulting from immune complex formation [20]. Incidentally, aldolase Ag brings us closer to the realization of such an anticipated target.

The IgM8A2 binding characteristics were investigated to find additional insights on the mAb. An indirect immunofluorescent assay revealed that IgM8A2 binds *T*. *congolense*, *T*. *b*. *brucei*, *T*. *evansi* and *T*. *vivax*. Cross-reactivity of IgM8A2 was not surprising, given that the *Tco*ALD primary sequence is identical to those of *T*. *b*. *brucei*, *T*. *evansi* and *T*. *vivax* [22]. However, it can be seen that the cross-reactivity of the IgM8A2 does not affect the specificity of the assay, as the latter is determined by the Nb (Ag-capture reagent). Perhaps, the sensitivity of a LFA for diagnosis of *T*. *congolense* developed from a cross-reactive IgM8A2 (Ag detection reagent) may be affected when confronted with *T*. *congolense* mix infection with *T*. *b*. *brucei*, *T*. *vivax*, or *T*. *evansi*. A practical solution is to impregnate large quantities of IgM8A2 into a conjugate pad, in order to sequester competing aldolases. On the other hand, a cross-reactive IgM8A2 would work well for a multiplex immunoassay for multi-species detection. In this case, a trypanosome species test line could be printed with a species-specific Nb, adopting a strategy reviewed in Anfossi *et al* [36]. It was also observed that denaturation of aldolase completely abolish detection by IgM8A2, suggesting the mAb binds a conformational epitope. Results of a competition ELISA, conducted to verify whether Nb474 and IgM8A2 compete for the same epitope, suggested nothing of that kind, as the binding of Nb474 to *Tco*ALD was not affected by IgM8A2 and *vice versa*. The heterologous binding site recognition of the two reagents solves the technical challenge, which affected the translation of Nb474 homologous sandwich ELISA into a LFA device.

Assessment of the Nb/mAb “hybrid” sandwich ELISA for cross-reactivity on naïve or trypanosome-infected mouse sera revealed the assay’s specificity for *T*. *congolense*, further reiterating the contribution of Nb (Ag capturing reagent) as a determinant of the assay’s specificity. To assess if, indeed, opportunity exists for translation of Nb/mAb “hybrid” sandwich ELISA prototype into a lateral flow assay, a dot blot employing IgM8A2-G was performed. IgM8A2-G specifically detected recombinant *Tco*ALD as well as *Tco*Lys, albeit with a stronger signal intensity on spots blotted with the recombinant protein. Taken together, *Au*-labelling did not affect the binding of IgM8A2 to aldolase. High signal intensity observed on recombinant *Tco*ALD compared to the lysate could have been influenced by variation in the Ag concentration, being higher in the recombinant protein preparation than in the lysate.

Given that the new technology has removed a bottle-neck encountered with the nanoparticle-labelling of Ag detection Nb, proliferation of Nb/mAb “hybrid” Ag detection tests for infectious diseases including African trypanosomiasis is envisaged. Hitherto, labelling of Nb with *Au* nanoparticles for incorporation as Ag detection reagent in a LFA has proven a daunting task. The latter is among the challenges that have hindered the progression of Nb-based test devices from development to commercialization. The current “hybrid” technology, in which detection Nb was replaced by a mAb, offers a relief to the obstacle. Furthermore, this innovation demonstrates how mAb could be exploited to synergize Nbs in the development of analytical devices and *vice versa*. The “hybrid” sandwich technology reported here, sets stage for rapid advancement of Nb-based LFA device development in all spheres. Construction of a LFA from the recently developed Nb/mAb “hybrid” solid-phase ELISA prototype reported should be first next step in this direction.

## Supporting information

S1 Materials and Methods Indirect immunofluorescence staining of trypanosome using IgM8A2-B as a primary antibody. (DOCX)

S2 Materials and Methods Assessment of binding competition between Nb474 and IgM8A2 by Competition ELISA-Indirect Antibody. (DOCX)

S1 Fig Assessment of antibody response in a set of mice immunized with recombinant *Tco*ALD by ELISA. (PDF)

S2 Fig Detection of ALD denatured at 55°C by Nb474H/IgM8A2 ELISA. (PDF)

S3 Fig A) Assessing the inhibition of Nb474 binding to *Tco*ALD with varying amounts of IgM8A2 B) Assessing the inhibition of IgM8A2 binding to *Tco*ALD with varying amounts of Nb474HA. (PDF)

S4 Fig Titration of *Tco*ALD to determine analytical sensitivity of the Nb474H/IgM8A2-B hybrid sandwich ELISA. (PDF)

S1 Table Setup of Nb474H coating across a 96-well ELISA plate. (DOCX)

S2 Table Setup of the IgM8A2-B dilutions dispensed across a 96-well plate row-wise. (DOCX)

S3 Table A layout of a 96-well ELISA plate filled with varying concentrations (μg/ml) of recombinant *Tco*ALD. (DOCX)

S4 Table A dot blot layout of a Gold-labelled IgM8A2 direct binding to *Tco*ALD. (DOCX)

S5 Table A dot blot layout of Nb474H/Gold-labelled IgM8A2 sandwich binding to *Tco*ALD. (DOCX)

S1 Data ELISA raw data obtained when hybridoma library was screened on *Tco*ALD. (XLS)

S2 Data ELISA raw data obtained when the thirteen hybridoma clones that scored positive on *Tco*ALD in the primary screening were re-screening on *Tco*ALD or irrelevant proteins. (XLS)

S3 Data ELISA raw data obtained when immunoglobulin isotype expressed by the hybridoma clones 1G9, 6C7, 7D9 and 8A2 was characterized. (XLSX)

S4 Data ELISA raw data obtained when IgM produced by hybridoma clones 1G9, 6C7, 7D9 and 8A2 were assessed, in a sandwich combination with Nb474H or Nb77, for binding *Tco*ALD or irrelevant protein. (XLS)

S5 Data ELISA raw data obtained when IgM8A2 was assessed, in a sandwich combination with Nb474H, for binding *T*. *congolense* lysate. (XLS)

S6 Data ELISA raw data obtained when the binding of strep-HRP to in vitro biotinylated IgM8A2 was assessed. (XLS)

S7 Data Raw data obtained when Nb474H/IgM8A2-B “hybrid” sandwich ELISA was assessed for specific recognition of *Tco*ALD. (XLS)

S8 Data ELISA raw data obtained following titration of capture Nb474H against detection IgM8A2-B for a matching concentration. (XLS)

S9 Data ELISA raw data obtained following evaluation of Nb474H/IgM8A2-B “hybrid” sandwich ELISA for detection of *T*. *congolense* infections. (XLS)

S10 Data Raw data obtained when mice immunized with *Tco*ALD were assessed by ELISA for antibody response against immunizing antigen. (XLS)

S11 Data Raw data obtained when the binding of Nb474H/IgM8A2-B “hybrid” sandwich ELISA to heat denatured *Tco*ALD was assessed. (XLS)

S12 Data ELISA raw data obtained following assessment of the inhibition of Nb474 binding to *Tco*ALD by IgM8A2. (XLS)

S13 Data ELISA raw data obtained following assessment of the inhibition of IgM8A2 binding to *Tco*ALD by Nb474. (XLS)

S14 Data ELISA raw data obtained when the limit of detection of *Tco*ALD by Nb474H/IgM8A2 hybrid sandwich ELISA was assessed. (XLS)
