# Supplementary material for: A Nanobody/Monoclonal Antibody “hybrid” sandwich technology offers an improved immunoassay strategy for detection of African trypanosome infections
Source: PLoS Negl Trop Dis. 2024 Jul 1;18(7):e0012294. doi: 10.1371/journal.pntd.0012294 (PMC11244815; doi:10.1371/journal.pntd.0012294)
Supplement: S1 Materials and Methods — (DOCX) [file pntd.0012294.s001.docx]

**S1 Materials and Methods. Indirect immunofluorescence staining of trypanosome using IgM8A2-B primary antibody**

**Procedure**

1. Trypanosomes propagated in mice were purified and the cell suspension in the phosphate saline glucose (PSG) solution (NaCl, 36.5 mM; NaH_2_PO_4_, 3.6 mM; Na_2_HPO_4_, 59.5 mM; Glucose, 88.8 mM) was pelleted by centrifugation (16,000 g, 9 mins, 22°C).
2. The pellet was resuspended in 320 µL dilute formaldehyde (1% v/v) in 1x PBS for 10 min at 22°C.
3. 1M glycine dissolved in 1xPBS was added (40 µl) to the cell suspension and mixed by a pipette followed by incubation (10 mins, 22°C). Thereafter, PSG was added (800 µl) to dilute the fixed cell suspension.
4. The dilution was spotted (20 µl) onto a microscope slide (*MICROS SLIDES NewSilaneIII, MUTO PURE CHEMICALS CO. LTD, Cat. No. 5116-20F*) and left to air-dry. The slide was transferred into a humidified tray (*Simport*).
5. The spots were washed thrice by flooding with bovine serum albumin (100 µl) diluted to 1 mg/ml in 1xPBS.
6. A mouse IgM8A2-B or irrelevant commercial Ab *Biotin anti-mouse IgM Antibody* (*Biolegend, Cat. No. 406504/500 µg*) were each diluted to 2.5 µg/ml in 1000 µl *Ab diluent* [1mg/ml BSA in PBS containing 0.1% (v/v) Triton X-100]. Each of the diluted primary Abs or *Ab diluent* was added (200 µl) to the washed spots on the slide and incubation was continued in a humidified tray (1hr, 22°C).
7. The spotted primary antibody was washed thrice and a *Cy3 Streptavidin* (*Biolegend, Cat. No. 405215*) diluted to 1 µg/ml in *Ab diluent* was added (500 μl).
8. The slide was incubated in the dark (45 min, 22°C) and washed thrice with 1xPBS (500μl/spot).
9. The 4′,6-diamidino-2-phenylindole (DAPI) stock solution (1mg/ml) was diluted to a working concentration (1µg/ml) in 1xPBS and added (500 µl) to the washed slide.
10. The slide was kept in the dark for 3 mins.
11. The spot was washed thrice by flooding with 1xPBS (500 μl/spot) and the slide was waved in the air until it was dump dried.
12. Using P-1000 micropipette tip, a tiny drop of *VECTASHIELD® Antifade Mounting Media* (*Vector laboratories, Cat. No. H-1000*) was put on a coverslip and the face of the slide with trypanosome smear was laid on the cover slip with gentle pressing to ensure spreading of the antifade.
13. The slide was turned upside and the sides of the cover slip were shielded with a nail polish.
14. Finally, the preparation was examined by immunofluorescent microscope (OLYMPUS *IX83P2ZF*) under x 60 objective lens. Examination was conducted in both BLUE (for DAPI) and RED (for *Cy3 Streptavidin*) channel at exposure 60.98 ms using *OLYMPUS cellSens Dimension* *1.18 (Build 16686)* computer software.

***Summary of experimental steps***

| **Experiment** | **Steps** | | | |
| --- | --- | --- | --- | --- |
|  | 1. Spot trypanosomes | 1. Add primary Ab | 1. Add secondary Ab | 1. Examine the slides |
| **Control 1** | Spot trypanosome onto microscope slides | Ab diluent only | Cy3 Strep | Examine slide |
| **Control 2** | “ | Biotin anti-mouse IgM | “ | “ |
| **Test** | “ | Biotin IgM8A2 | “ | “ |

|  |  | Trypanosome species | | | | | | | | | | | |
| --- | --- | --- | --- | --- | --- | --- | --- | --- | --- | --- | --- | --- | --- |
|  |  | 1. *T. congolense* | | | **B)** *T. b. brucei* | | | **C)** *T. evansi* | | | **D)** *T. vivax* | | |
|  |  | BLUE (DAPI) | RED (Cy3 Strep) | Merge | BLUE (DAPI) | RED (Cy3 Strep) | Merge | BLUE (DAPI) | RED (Cy3 Strep) | Merge | BLUE (DAPI) | RED (Cy3 Strep) | Merge |
| Treatment | Control 1 (-) |  |  |  |  |  |  |  |  |  |  |  |  |
|  | Control 2 (-) |  |  |  |  |  |  |  |  |  |  |  |  |
|  | Test (+/-) |  |  |  |  |  |  |  |  |  |  |  |  |
