# Supplementary material for: A Nanobody/Monoclonal Antibody “hybrid” sandwich technology offers an improved immunoassay strategy for detection of African trypanosome infections
Source: PLoS Negl Trop Dis. 2024 Jul 1;18(7):e0012294. doi: 10.1371/journal.pntd.0012294 (PMC11244815; doi:10.1371/journal.pntd.0012294)
Supplement: S2 Materials and Methods — (DOCX) [file pntd.0012294.s002.docx]

**S2 Materials and Methods. Assessment of binding competition between Nb474 and IgM8A2 by Competition ELISA-Indirect Antibody**

1. *Assessing the interference of IgM8A2 with the binding of Nb474HA to TcoALD*

**Procedure:**

1. Nb474H capture reagent was diluted and uniformly coated into wells (0.25 µg/well) of a 96well ELISA plate (**Table 1**). The coating was incubated for an overnight at 4°C.

**Table 1.** A layout of a 96-well ELISA plate indicating wells (shaded) filled with the Nb474H coating reagent.


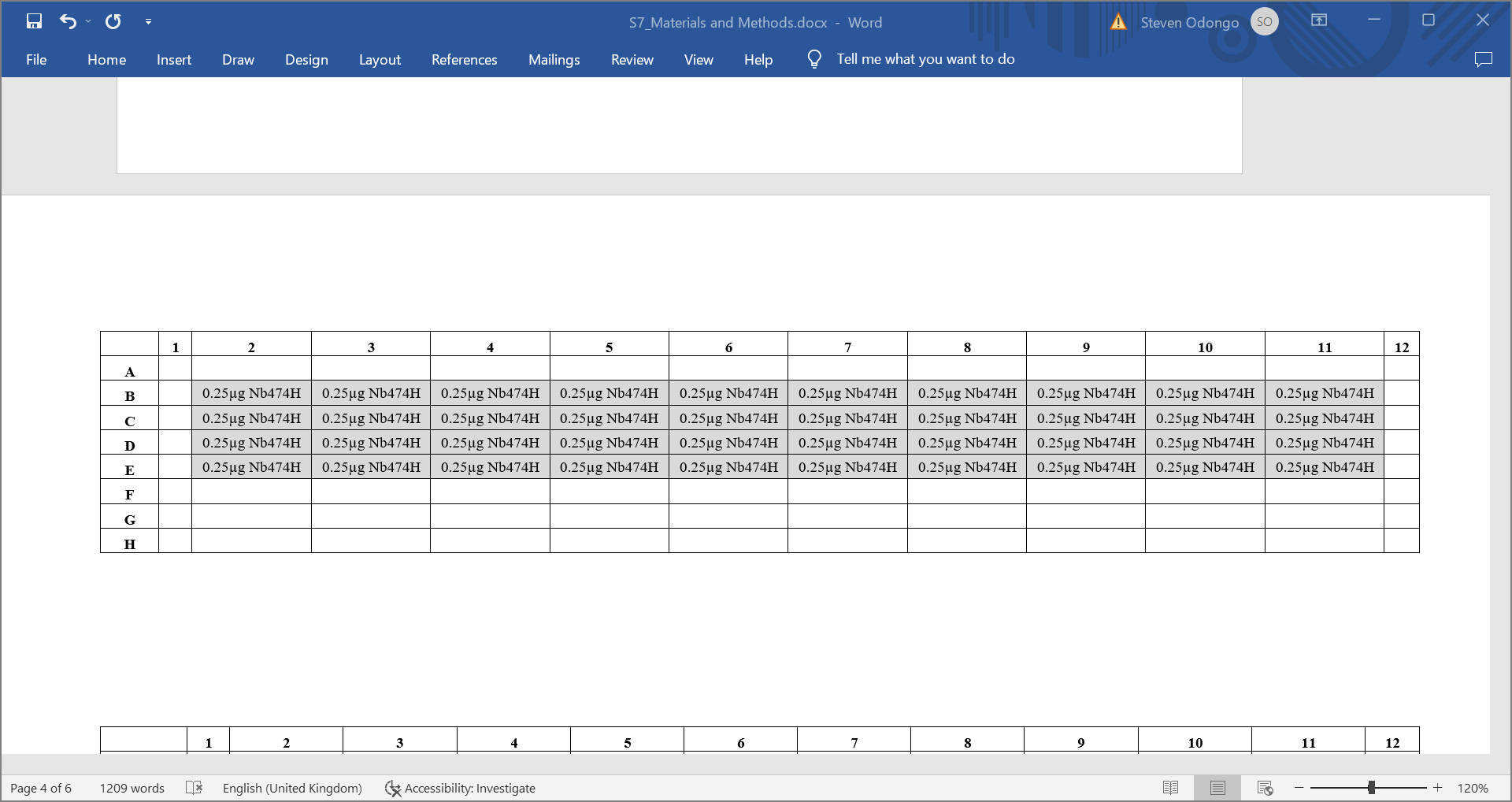


1. The next day coating was emptied and the well was washed thrice.
2. The washed well was blocked with *SuperBlock™* solution (160 µl/well) for 2 hrs with buffer refreshment after an hour.
3. The blocking buffer was emptied and the wells were washed thrice followed by addition of *Tco*ALD into the wells (0.5 µg/well) as shown (**Table 2**).

**Table 2.** A layout of a 96-well ELISA plate indicating wells (shaded) filled with *Tco*ALD solution or 1xPBS.


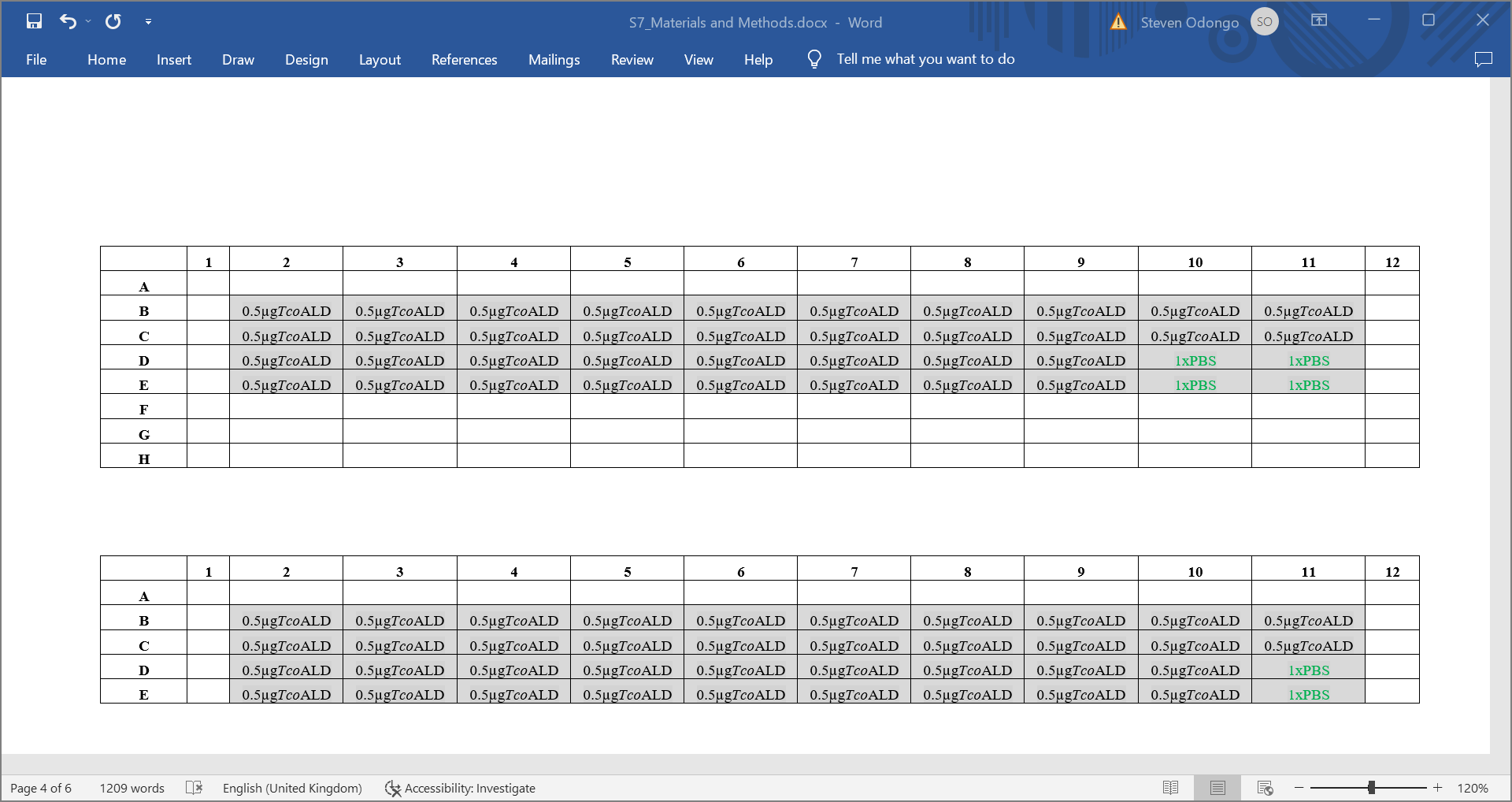


1. The binding reaction between *Tco*ALD and the Nb474H capture reagent proceeded for an hour at 22°C before the wells were washed thrice.
2. Thereafter, a constant amount of Nb474HA (0.25 µg) was mixed with a varying (two-fold dilution) of unlabeled IgM8A2 (ranging from 28 to 4.3x10^-4^ µg). The Nb474HA-IgM8A2 mixture was dispensed into the wells of the washed 96-well ELISA plate (**Table 3**). *NB: The* *wells D9-11, E9-11 which served as control each received “clean” Nb474HA (0.25 µg/ml).*

**Table 3.** A layout of a 96-well ELISA plate indicating wells (shaded) filled with Nb474HA-unlabelled IgM8A2 mixture.


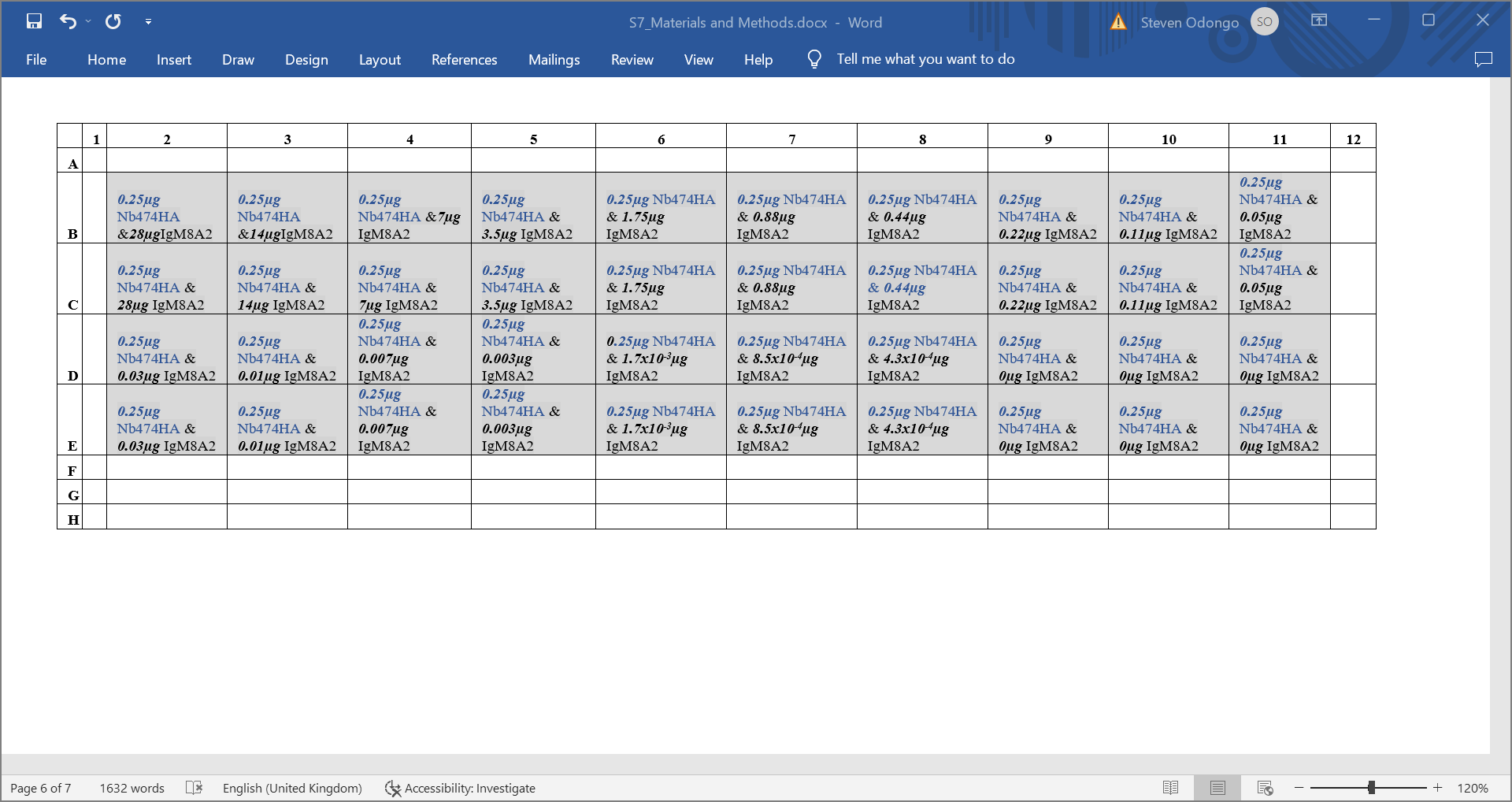


1. Afterwards, the binding reaction between the species of detection reagents (Nb474HA and IgM8A2), assessed for a binding competition, was allowed for an hour at 22°C.
2. The wells were washed four times. Then rat Anti-HA-Biotin (*sigma-aldrich, Cat. No. 12158167001*) was diluted in blocking buffer to a working concentration (**100 ng/ml)** and added (**50 μl/well**) into the wells. Incubation was allowed for an hour at 22°C.
3. The wells were washed four times and Strep-HRP solution diluted in blocking buffer (0.5 µg/ml) was added to the wells (50 µl/well) followed by incubation for an hour at 22°C.
4. The wells were washed five times and TMB was added followed by incubation in the dark for 15 mins at 22°C.
5. The reaction was stopped by adding 1M H_2_SO_4_ (50 µl/well) and the OD was read at 450nm.
6. *Assessing the interference of Nb474HA with the binding of IgM8A2 to TcoALD*

**Procedure:**

1. A 96-well ELISA plate was coated with Nb474H (0.25 µg/well) on the wells shown (**Table 4**).

**Table 4.** A layout of a 96-well ELISA plate indicating wells (shaded) filled with the Nb474H coating reagent.


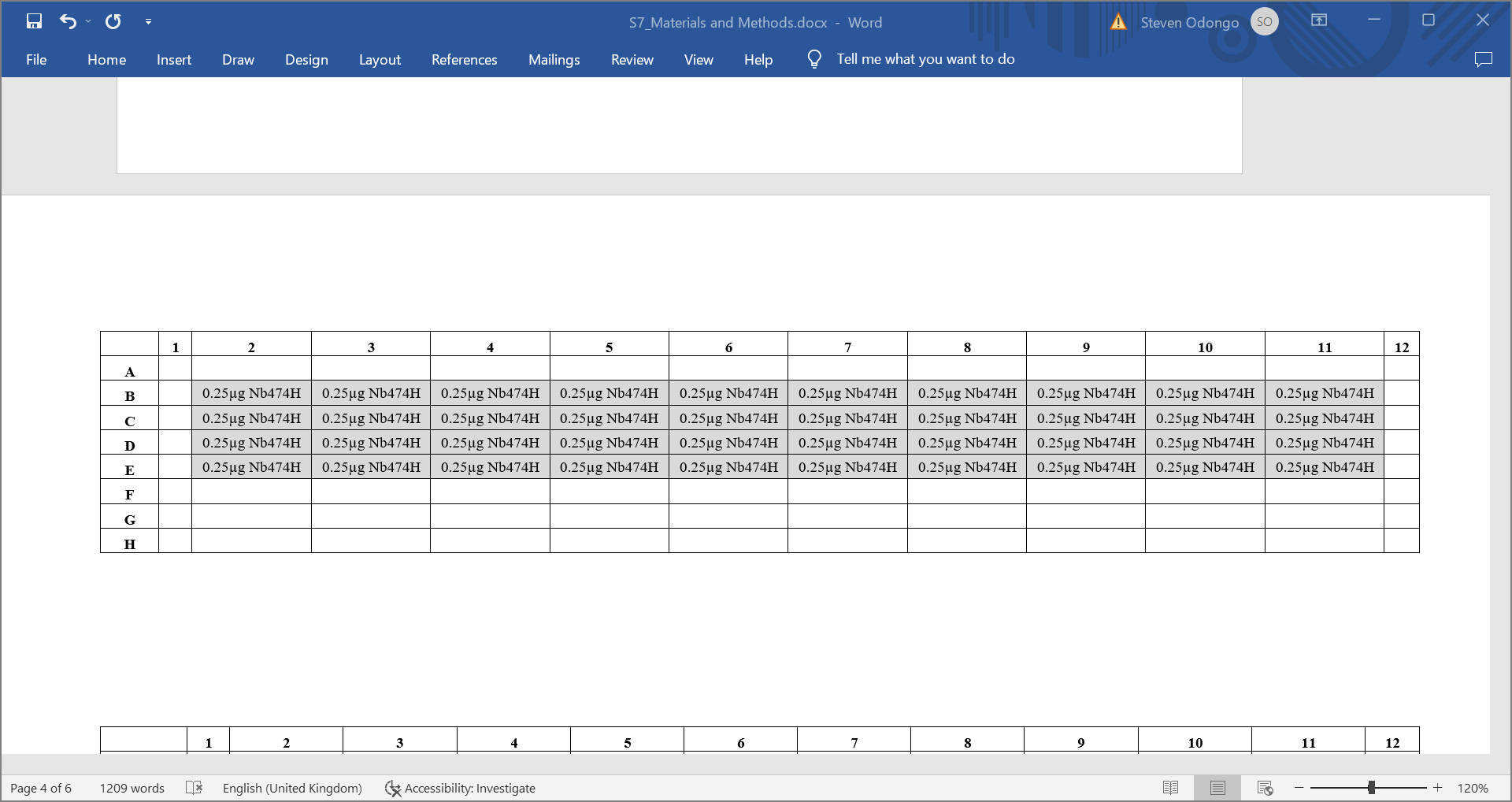


1. The coating was incubated on the plate for an overnight at 4°C.
2. The next day, wells were emptied of the unbound coating reagent and washed thrice.
3. Afterward, empty spaces in the washed wells were blocked with *SuperBlock™* solution (160 µl/well) at 22°C for 2 hours. A buffer refreshment was done after an hour.
4. Next, the blocking was discarded and the wells were washed thrice followed by addition of *Tco*ALD (0.5 µg/well) or 1x PBS only (**Table 5**).

**Table 5.** A layout of a 96-well ELISA plate indicating wells (shaded) filled with *Tco*ALD solution or 1xPBS


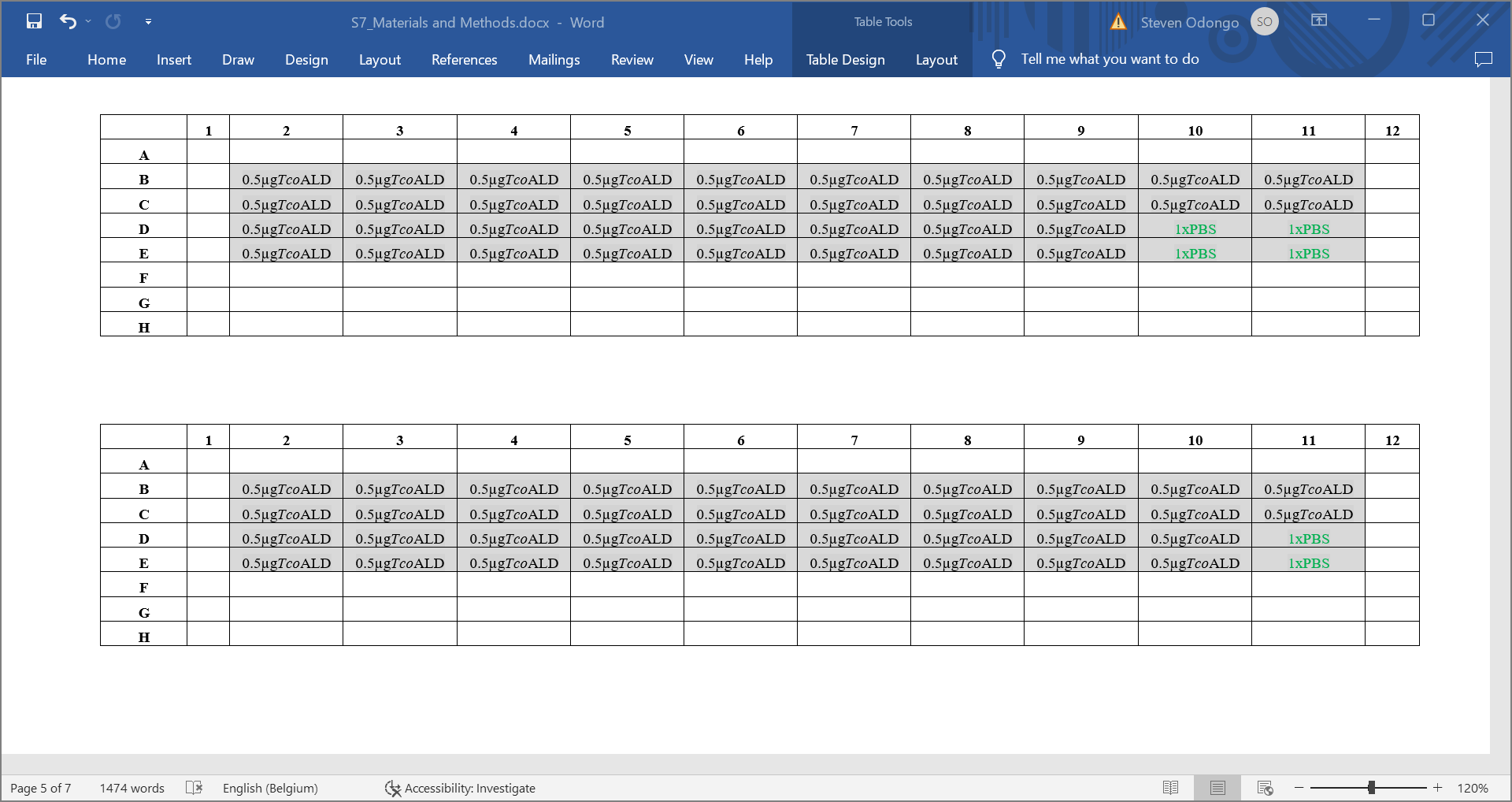


1. After an hour of incubation to allow the binding of *Tco*ALD to a coated capture reagent, the wells were washed thrice.
2. Then the IgM8A2 solution amount (0.22 µg/well) mixed with a two-fold decreasing concentration of Nb474HA (109.6 to 8.0x10^-4^ µg/well) was added into the wells (**Table 6**).

**Table 6.** A layout of a 96-well ELISA plate indicating wells (shaded) filled with unlabelled IgM8A2-Nb474HA mixture.


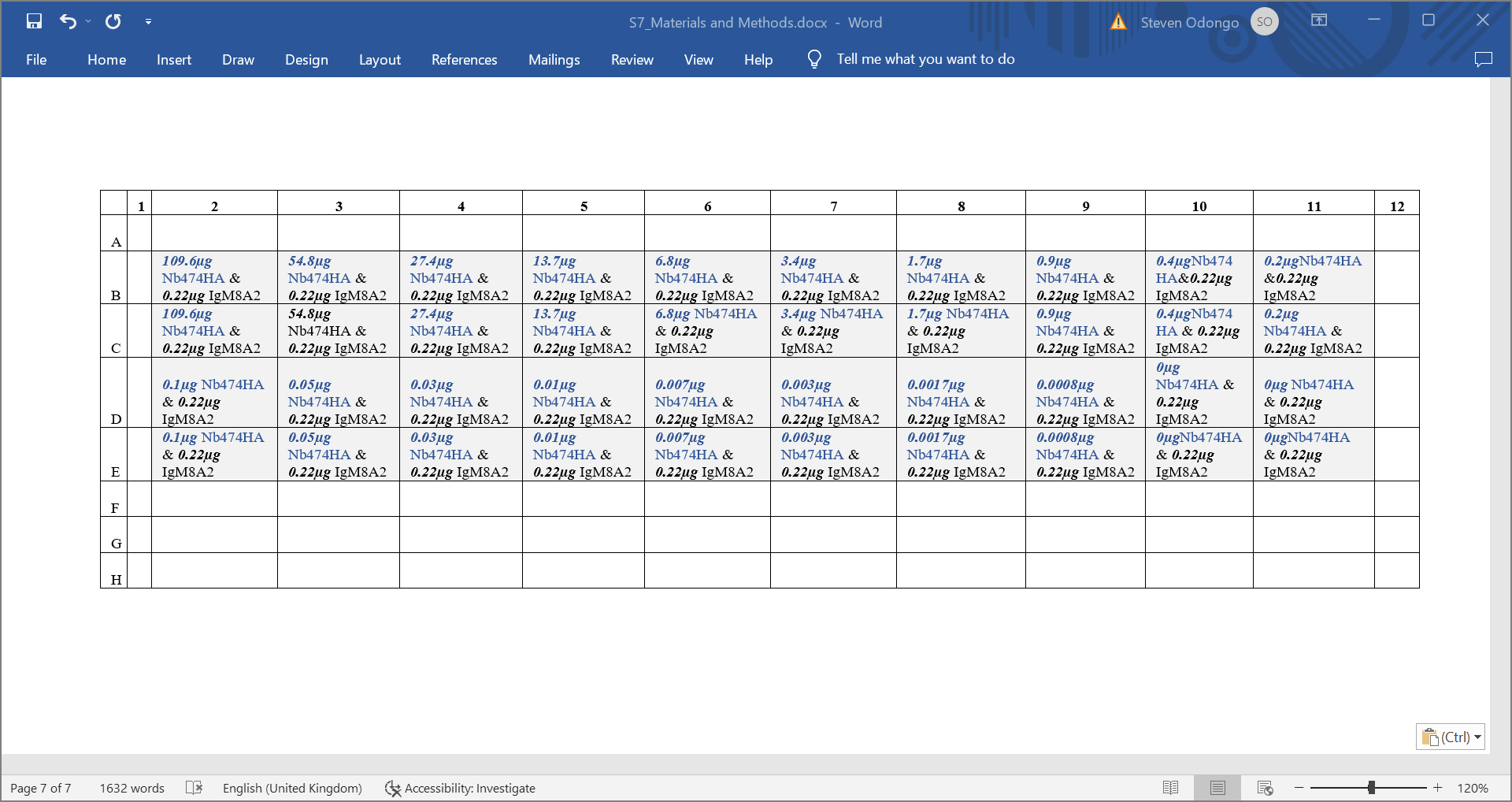


1. Afterwards, the binding reaction between the species of detection reagents (Nb474HA and IgM8A2), assessed for a binding competition, was allowed for an hour at 22°C.
2. Then goat anti-mouse IgM HRP diluted in the blocking buffer (1/1000) was added into the reaction wells (50 µl/well) and incubated for an hour at 22°C.
3. The wells were washed five times. Next, TMB substrate was added into the wells (50 µl/well) followed by incubation in the dark for 15 mins.
4. Thereafter, the reaction was stopped by adding 1M H_2_SO_4_ (50 µl/well) and OD was read at 450nm.
