# Supplementary figures and images for: A Nanobody/Monoclonal Antibody “hybrid” sandwich technology offers an improved immunoassay strategy for detection of African trypanosome infections
Source: PLoS Negl Trop Dis. 2024 Jul 1;18(7):e0012294. doi: 10.1371/journal.pntd.0012294 (PMC11244815; doi:10.1371/journal.pntd.0012294)

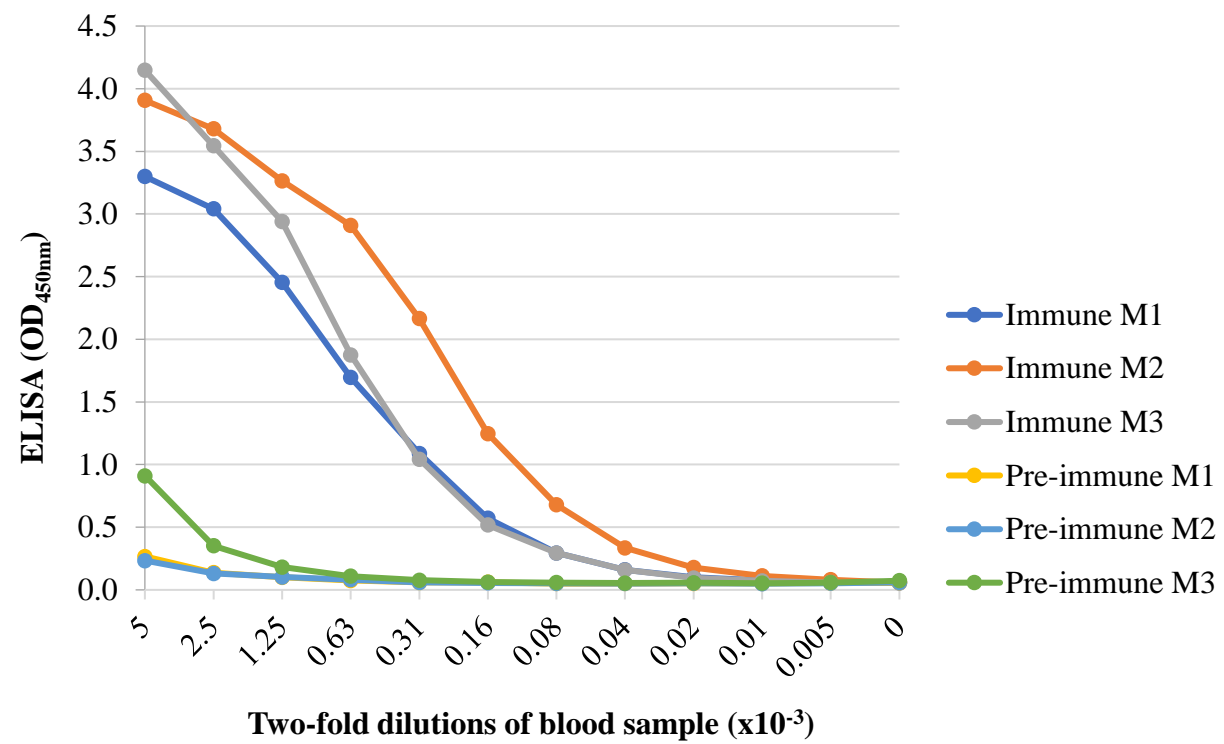

Supplement: S1 Fig — (PDF) [file pntd.0012294.s003.pdf]

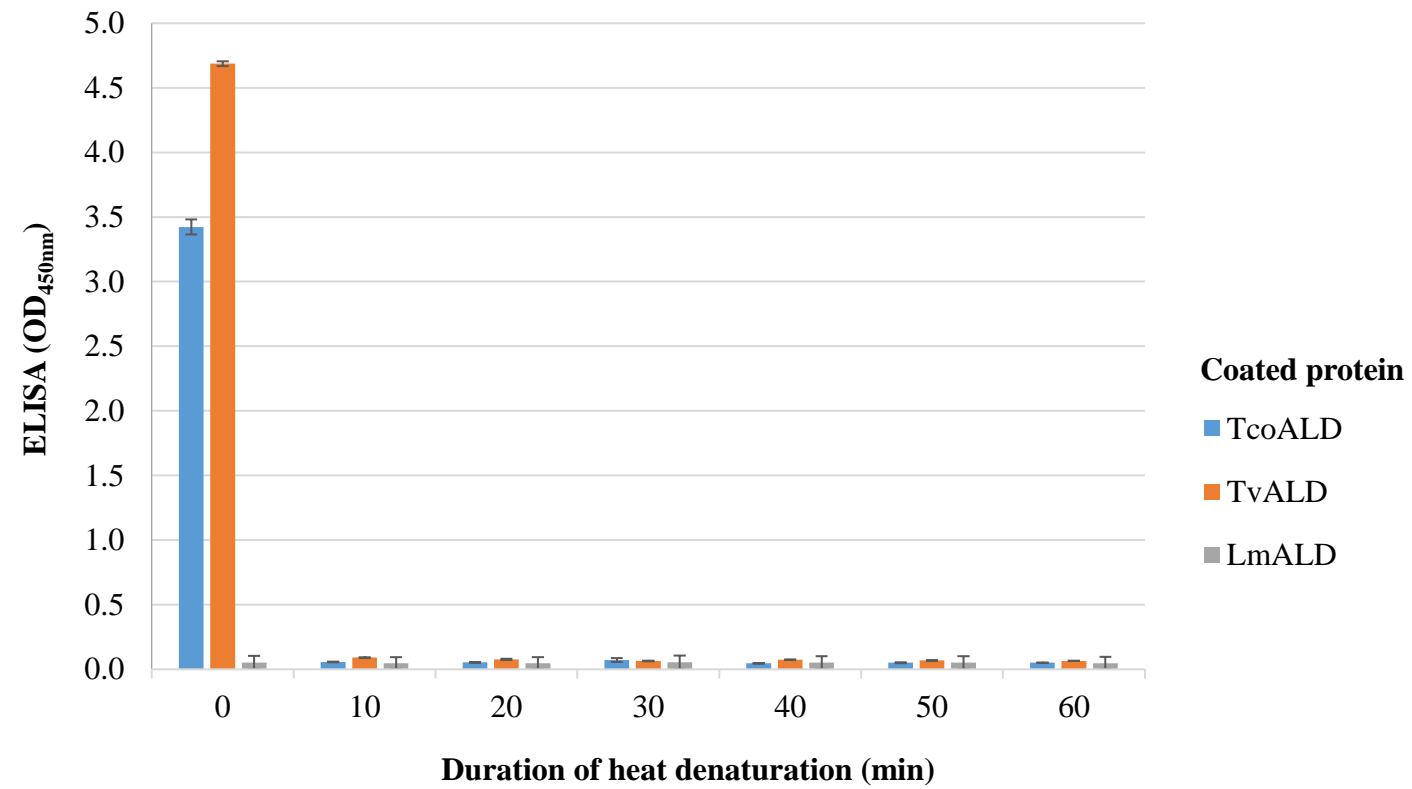

Supplement: S2 Fig — (PDF) [file pntd.0012294.s004.pdf]

**A**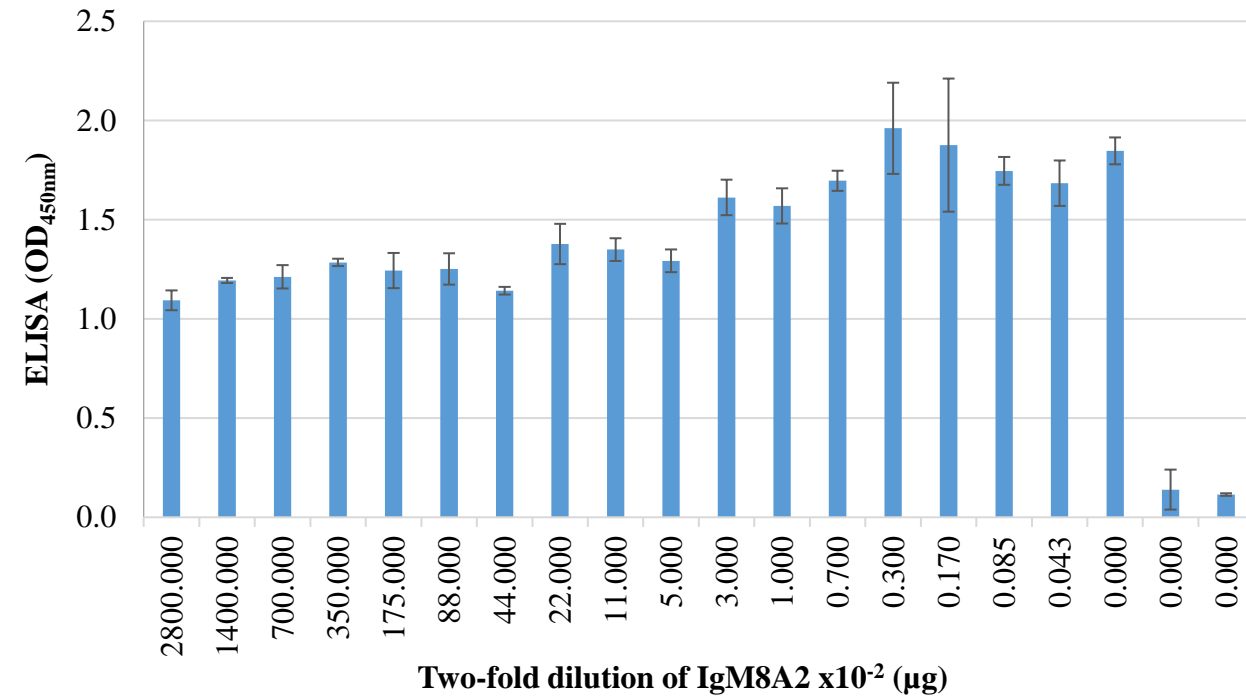**B**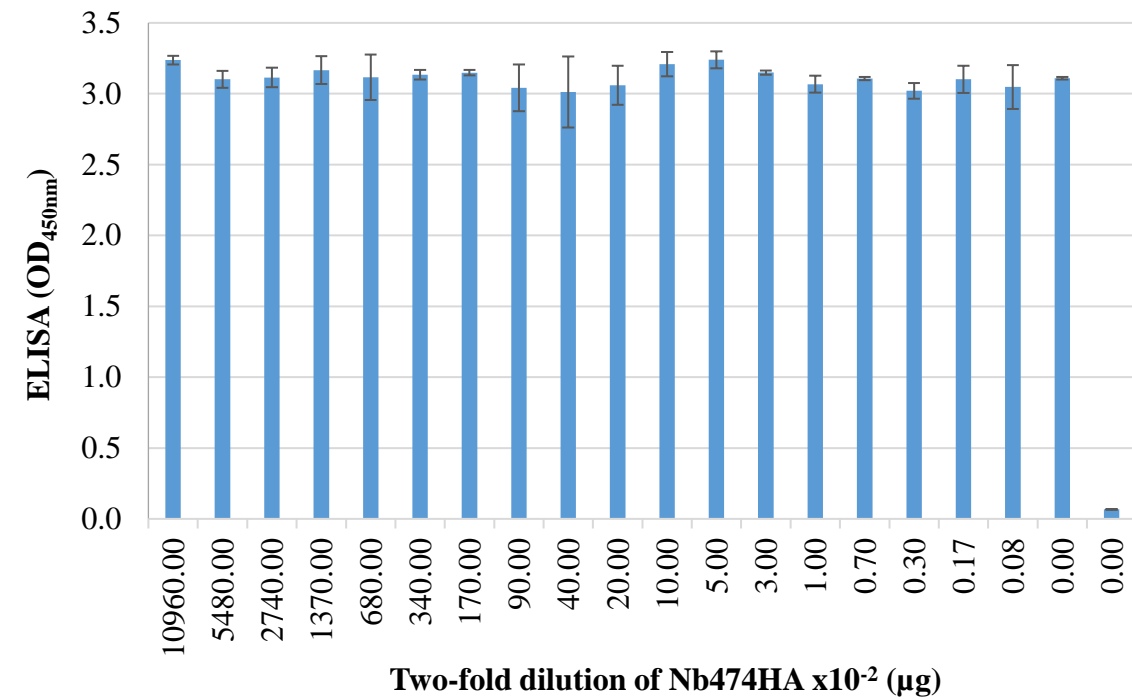

Supplement: S3 Fig — (PDF) [file pntd.0012294.s005.pdf]

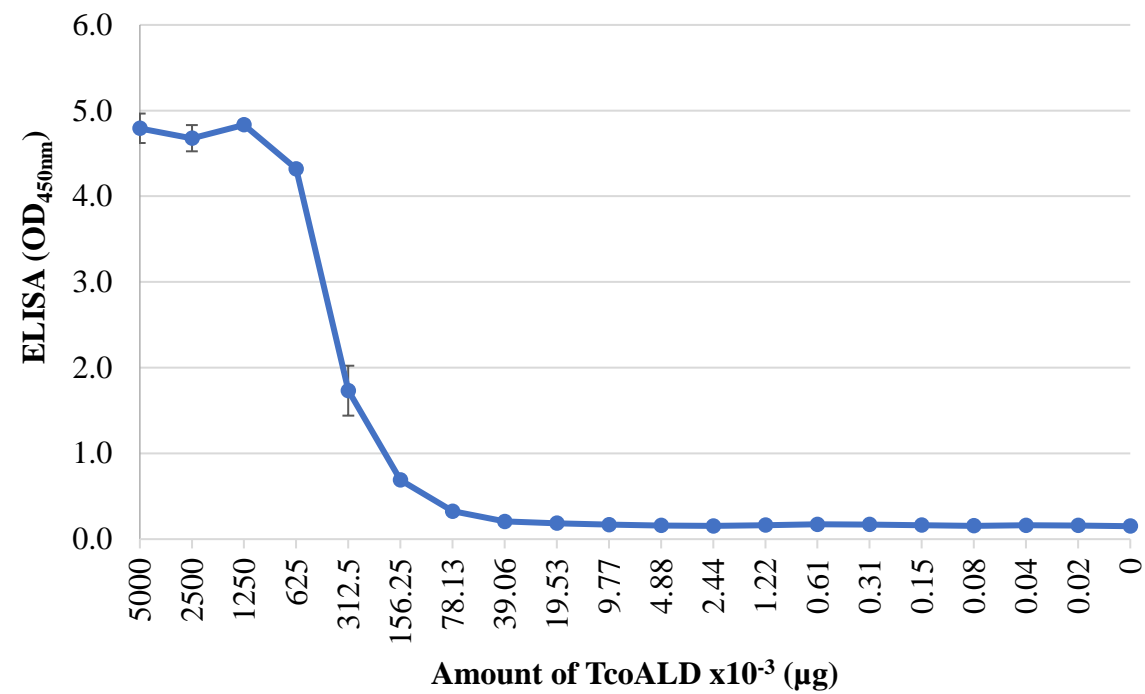

Supplement: S4 Fig — (PDF) [file pntd.0012294.s006.pdf]
