# Supplementary material for: A Nanobody/Monoclonal Antibody “hybrid” sandwich technology offers an improved immunoassay strategy for detection of African trypanosome infections
Source: PLoS Negl Trop Dis. 2024 Jul 1;18(7):e0012294. doi: 10.1371/journal.pntd.0012294 (PMC11244815; doi:10.1371/journal.pntd.0012294)
Supplement: S1 Table — (DOCX) [file pntd.0012294.s007.docx]

**S1 Table. Setup of Nb474H coating across a 96-well ELISA plate.** A serially diluted Nb474H was coated column-wise across a 96-well ELISA plate. Wells on column 1 were filled with the most concentrated sample (5µg/mL in 50µL/well) and those on column 11 received the least concentrated sample (0.005 µg/mL in 50 µL/well). Column 12 received 1xPBS only. The heatmap (color gradient) shows a decreasing concentration of Nb474H from red (most concentrated) to green (least concentrated).

**
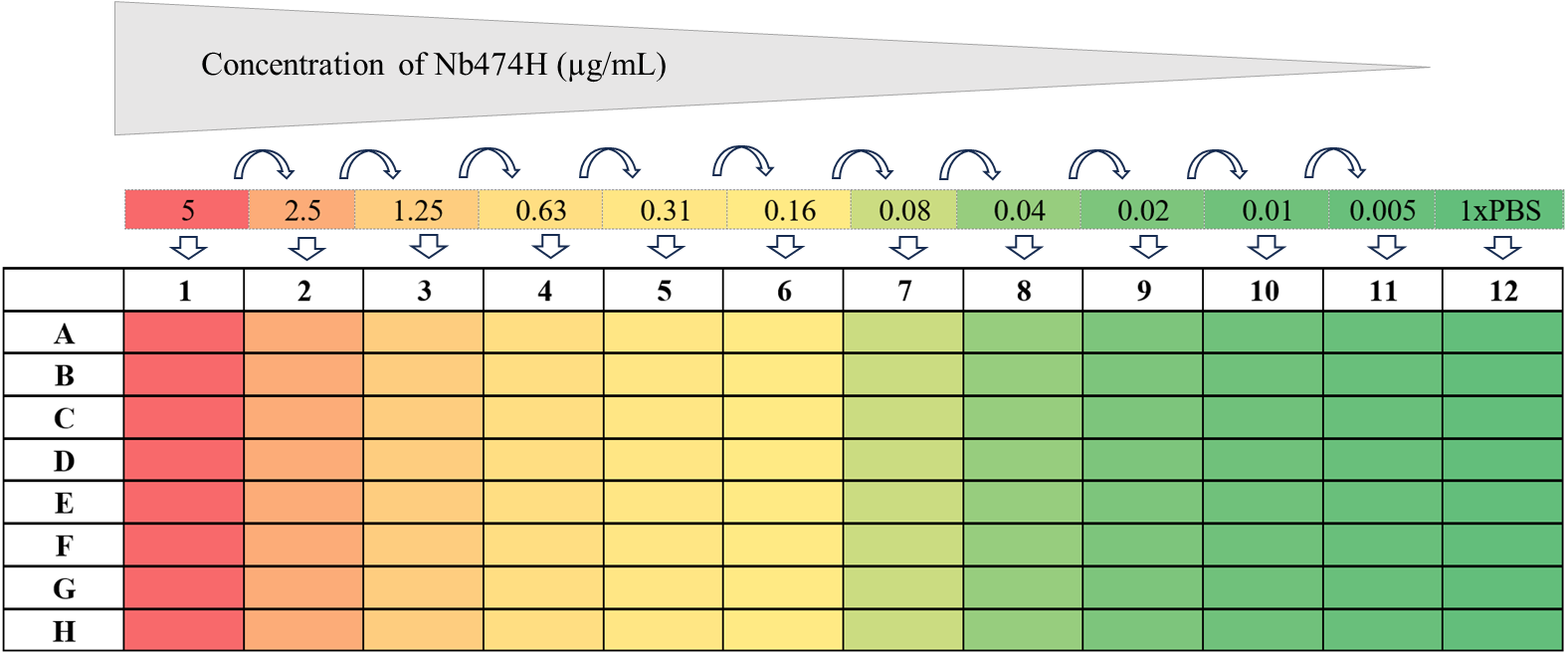
**

**S1 Table. Concentration (µg/mL) of Nb474H coated across a 96-well plate for a checker board titration**

| **Value** | **1** | **2** | **3** | **4** | **5** | **6** | **7** | **8** | **9** | **10** | **11** | **12** |
| --- | --- | --- | --- | --- | --- | --- | --- | --- | --- | --- | --- | --- |
| **A** | 5 µg | 2.5 µg | 1.25 µg | 0.63 µg | 0.31 µg | 0.16 µg | 0.08 µg | 0.04 µg | 0.02 µg | 0.01 µg | 0.005 µg | 1xPBS |
| **B** | 5 µg | 2.5 µg | 1.25 µg | 0.63 µg | 0.31 µg | 0.16 µg | 0.08 µg | 0.04 µg | 0.02 µg | 0.01 µg | 0.005 µg | 1xPBS |
| **C** | 5 µg | 2.5 µg | 1.25 µg | 0.63 µg | 0.31 µg | 0.16 µg | 0.08 µg | 0.04 µg | 0.02 µg | 0.01 µg | 0.005 µg | 1xPBS |
| **D** | 5 µg | 2.5 µg | 1.25 µg | 0.63 µg | 0.31 µg | 0.16 µg | 0.08 µg | 0.04 µg | 0.02 µg | 0.01 µg | 0.005 µg | 1xPBS |
| **E** | 5 µg | 2.5 µg | 1.25 µg | 0.63 µg | 0.31 µg | 0.16 µg | 0.08 µg | 0.04 µg | 0.02 µg | 0.01 µg | 0.005 µg | 1xPBS |
| **F** | 5 µg | 2.5 µg | 1.25 µg | 0.63 µg | 0.31 µg | 0.16 µg | 0.08 µg | 0.04 µg | 0.02 µg | 0.01 µg | 0.005 µg | 1xPBS |
| **G** | 5 µg | 2.5 µg | 1.25 µg | 0.63 µg | 0.31 µg | 0.16 µg | 0.08 µg | 0.04 µg | 0.02 µg | 0.01 µg | 0.005 µg | 1xPBS |
| **H** | 5 µg | 2.5 µg | 1.25 µg | 0.63 µg | 0.31 µg | 0.16 µg | 0.08 µg | 0.04 µg | 0.02 µg | 0.01 µg | 0.005 µg | 1xPBS |

The dilutions of Nb474H were coated column-wise. Wells on column 1 received the least dilute sample (5µg/mL in 50µL/well) going on until wells in column 11, which received the most dilute sample (0.005 µg/mL in 50 µL/well). The wells in column 12 received 1xPBS only.

| 5 | 2.5 | 1.25 | 0.63 | 0.31 | 0.16 | 0.08 | 0.04 | 0.02 | 0.01 | 0.005 | 1xPBS |
| --- | --- | --- | --- | --- | --- | --- | --- | --- | --- | --- | --- |

|  | **1** | **2** | **3** | **4** | **5** | **6** | **7** | **8** | **9** | **10** | **11** | **12** |
| --- | --- | --- | --- | --- | --- | --- | --- | --- | --- | --- | --- | --- |
| **A** |  |  |  |  |  |  |  |  |  |  |  |  |
| **B** |  |  |  |  |  |  |  |  |  |  |  |  |
| **C** |  |  |  |  |  |  |  |  |  |  |  |  |
| **D** |  |  |  |  |  |  |  |  |  |  |  |  |
| **E** |  |  |  |  |  |  |  |  |  |  |  |  |
| **F** |  |  |  |  |  |  |  |  |  |  |  |  |
| **G** |  |  |  |  |  |  |  |  |  |  |  |  |
| **H** |  |  |  |  |  |  |  |  |  |  |  |  |
