# Supplementary material for: A Nanobody/Monoclonal Antibody “hybrid” sandwich technology offers an improved immunoassay strategy for detection of African trypanosome infections
Source: PLoS Negl Trop Dis. 2024 Jul 1;18(7):e0012294. doi: 10.1371/journal.pntd.0012294 (PMC11244815; doi:10.1371/journal.pntd.0012294)
Supplement: S2 Table — (DOCX) [file pntd.0012294.s008.docx]

**S2 Table. Setup of the IgM8A2-B dilutions dispensed across a 96-well plate row-wise.** IgM8A2-B was added into wells across the plate by concentration in that row A received the most concentrated sample (5 µg/mL in 50 µL/well) until G, which received the least concentrated sample (0.08 µg/mL in 50 µL/well). The wells on row H, which served as negative control received 1x PBS only (50 µL/well). The heatmap (color gradient) indicate decreasing concentration of IgM8A2-B from red (most concentrated) to green (least concentrated).


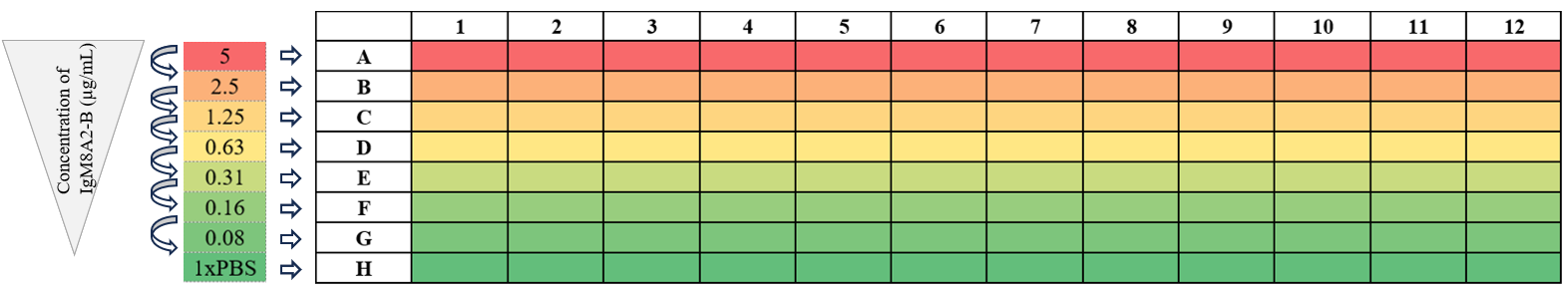


**S2 Table. IgM8A2-B serial dilutions added row-wise across a 96-well plate during a checker board titration**

| **Value** | **1** | **2** | **3** | **4** | **5** | **6** | **7** | **8** | **9** | **10** | **11** | **12** |
| --- | --- | --- | --- | --- | --- | --- | --- | --- | --- | --- | --- | --- |
| **A** | 05µg/mL | 05µg/mL | 05µg/mL | 05µg/mL | 05µg/mL | 05µg/mL | 05µg/mL | 05µg/mL | 05µg/mL | 05µg/mL | 05µg/mL | 05µg/mL |
| **B** | 2.5µg/mL | 2.5µg/mL | 2.5µg/mL | 2.5µg/mL | 2.5µg/mL | 2.5µg/mL | 2.5µg/mL | 2.5µg/mL | 2.5µg/mL | 2.5µg/mL | 2.5µg/mL | 2.5µg/mL |
| **C** | 1.25µg/mL | 1.25µg/mL | 1.25µg/mL | 1.25µg/mL | 1.25µg/mL | 1.25µg/mL | 1.25µg/mL | 1.25µg/mL | 1.25µg/mL | 1.25µg/mL | 1.25µg/mL | 1.25µg/mL |
| **D** | 0.63µg/mL | 0.63µg/mL | 0.63µg/mL | 0.63µg/mL | 0.63µg/mL | 0.63µg/mL | 0.63µg/mL | 0.63µg/mL | 0.63µg/mL | 0.63µg/mL | 0.63µg/mL | 0.63µg/mL |
| **E** | 0.31µg/mL | 0.31µg/mL | 0.31µg/mL | 0.31µg/mL | 0.31µg/mL | 0.31µg/mL | 0.31µg/mL | 0.31µg/mL | 0.31µg/mL | 0.31µg/mL | 0.31µg/mL | 0.31µg/mL |
| **F** | 0.16µg/mL | 0.16µg/mL | 0.16µg/mL | 0.16µg/mL | 0.16µg/mL | 0.16µg/mL | 0.16µg/mL | 0.16µg/mL | 0.16µg/mL | 0.16µg/mL | 0.16µg/mL | 0.16µg/mL |
| **G** | 0.08µg/mL | 0.08µg/mL | 0.08µg/mL | 0.08µg/mL | 0.08µg/mL | 0.08µg/mL | 0.08µg/mL | 0.08µg/mL | 0.08µg/mL | 0.08µg/mL | 0.08µg/mL | 0.08µg/mL |
| **H** | 1xPBS | 1xPBS | 1xPBS | 1xPBS | 1xPBS | 1xPBS | 1xPBS | 1xPBS | 1xPBS | 1xPBS | 1xPBS | 1xPBS |

When IgM8A2-B was added into wells, row A received the least diluted sample (5 µg/mL in 50 µL/well) going until G, which received the highest dilution (0.08 µg/mL in 50 µL/well). The wells in row H, which served as negative control received 1x PBS only (50 µL/well).

|  | **1** | **2** | **3** | **4** | **5** | **6** | **7** | **8** | **9** | **10** | **11** | **12** |
| --- | --- | --- | --- | --- | --- | --- | --- | --- | --- | --- | --- | --- |
| **A** | 5 | 5 | 5 | 5 | 5 | 5 | 5 | 5 | 5 | 5 | 5 | 5 |
| **B** | 2,5 | 2,5 | 2,5 | 2,5 | 2,5 | 2,5 | 2,5 | 2,5 | 2,5 | 2,5 | 2,5 | 2,5 |
| **C** | 1,25 | 1,25 | 1,25 | 1,25 | 1,25 | 1,25 | 1,25 | 1,25 | 1,25 | 1,25 | 1,25 | 1,25 |
| **D** | 0,63 | 0,63 | 0,63 | 0,63 | 0,63 | 0,63 | 0,63 | 0,63 | 0,63 | 0,63 | 0,63 | 0,63 |
| **E** | 0,31 | 0,31 | 0,31 | 0,31 | 0,31 | 0,31 | 0,31 | 0,31 | 0,31 | 0,31 | 0,31 | 0,31 |
| **F** | 0,16 | 0,16 | 0,16 | 0,16 | 0,16 | 0,16 | 0,16 | 0,16 | 0,16 | 0,16 | 0,16 | 0,16 |
| **G** | 0,08 | 0,08 | 0,08 | 0,08 | 0,08 | 0,08 | 0,08 | 0,08 | 0,08 | 0,08 | 0,08 | 0,08 |
| **H** | 0 | 0 | 0 | 0 | 0 | 0 | 0 | 0 | 0 | 0 | 0 | 0 |
