# Supplementary material for: A Nanobody/Monoclonal Antibody “hybrid” sandwich technology offers an improved immunoassay strategy for detection of African trypanosome infections
Source: PLoS Negl Trop Dis. 2024 Jul 1;18(7):e0012294. doi: 10.1371/journal.pntd.0012294 (PMC11244815; doi:10.1371/journal.pntd.0012294)
Supplement: S3 Table — (DOCX) [file pntd.0012294.s009.docx]

**S3 Table. A layout of a 96-well ELISA plate filled with varying concentrations (µg/mL) of recombinant *Tco*ALD.** Each of the dilution sets was dispensed (50 µL/well) in duplicate. Wells B2&C2 each received 100 µg/mL sample going on to wells B11&C11, which each received 0.195 µg/mL sample. Then wells D2&E3 each received sample diluted to 0.098 µg/mL progressing on to wells D10&E10, which each received 0.0004 µg/mL. The last duplicate wells D11&E11 each received 1xPBS only. Heatmap (color gradient) shows a decreasing concentration gradient from red (the most concentrated) to green (the least concentrated).

|  | **1** | **2** | **3** | **4** | **5** | **6** | **7** | **8** | **9** | **10** | **11** | **12** |
| --- | --- | --- | --- | --- | --- | --- | --- | --- | --- | --- | --- | --- |
| **A** |  |  |  |  |  |  |  |  |  |  |  |  |
| **B** |  | 100 | 50 | 25 | 12.5 | 6.25 | 3.13 | 1.56 | 0.78 | 0.39 | 0.2 |  |
| **C** |  | 100 | 50 | 25 | 12.5 | 6.25 | 3.13 | 1.56 | 0.78 | 0.39 | 0.2 |  |
| **D** |  | 0.1 | 0.05 | 0.02 | 0.01 | 0.006 | 0.003 | 0.0015 | 0.0008 | 0.0004 | 1xPBS |  |
| **E** |  | 0.1 | 0.05 | 0.02 | 0.01 | 0.006 | 0.003 | 0.0015 | 0.0008 | 0.0004 | 1xPBS |  |
| **F** |  |  |  |  |  |  |  |  |  |  |  |  |
| **G** |  |  |  |  |  |  |  |  |  |  |  |  |
| **H** |  |  |  |  |  |  |  |  |  |  |  |  |

**S3 Table. A serial dilution (two-fold) of recombinant *Tco*ALD protein dispensed across a 96-well plate to determine the detection limit (analytical sensitivity) by Nb474/IgM8A2-B hybrid sandwich system**

| **Value** | **1** | **2** | **3** | **4** | **5** | **6** | **7** | **8** | **9** | **10** | **11** | **12** |
| --- | --- | --- | --- | --- | --- | --- | --- | --- | --- | --- | --- | --- |
| **A** |  |  |  |  |  |  |  |  |  |  |  |  |
| **B** |  | 100 µg/mL | 50 µg/mL | 25 µg/mL | 12.5 µg/mL | 6.25 µg/mL | 3.13 µg/mL | 1.56 µg/mL | 0.78 µg/mL | 0.39 µg/mL | 0.20 µg/mL |  |
| **C** |  | 100 µg/mL | 50 µg/mL | 25 µg/mL | 12.5 µg/mL | 6.25 µg/mL | 3.13 µg/mL | 1.56 µg/mL | 0.78 µg/mL | 0.39 µg/mL | 0.20 µg/mL |  |
| **D** |  | 0.1 µg/mL | 0.05 µg/mL | 0.02 µg/mL | 0.01 µg/mL | 0.006 µg/mL | 0.003 µg/mL | 0.0015 µg/mL | 0.0008 µg/mL | 0.0004 µg/mL | 1xPBS |  |
| **E** |  | 0.1 µg/mL | 0.05 µg/mL | 0.02 µg/mL | 0.01 µg/mL | 0.006 µg/mL | 0.003 µg/mL | 0.0015 µg/mL | 0.0008 µg/mL | 0.0004 µg/mL | 1xPBS |  |
| **F** |  |  |  |  |  |  |  |  |  |  |  |  |
| **G** |  |  |  |  |  |  |  |  |  |  |  |  |
| **H** |  |  |  |  |  |  |  |  |  |  |  |  |

The dilutions of IgM8A2-B (100 – 4x10^-4^ µg/mL) were added (50 µL/well) in duplicate. The wells, B2&C2, each received 100 µg/mL sample going onto wells, B11&C11, each received 0.195 µg/mL sample and back to wells, D2&E3, each received 0.098 µg/mL sample and progressing until reaching the wells, D10E&10, which each received 0.0004µg/mL. The last duplicate wells D11&E11, each received 1xPBS only.

|  | **1** | **2** | **3** | **4** | **5** | **6** | **7** | **8** | **9** | **10** | **11** | **12** |
| --- | --- | --- | --- | --- | --- | --- | --- | --- | --- | --- | --- | --- |
| **A** |  |  |  |  |  |  |  |  |  |  |  |  |
| **B** |  | 100 | 50 | 25 | 12,5 | 6,25 | 3,13 | 1,56 | 0,78 | 0,39 | 0,20 |  |
| **C** |  | 100 | 50 | 25 | 12,5 | 6,25 | 3,13 | 1,56 | 0,78 | 0,39 | 0,20 |  |
| **D** |  | 0,1 | 0,05 | 0,02 | 0,01 | 0,006 | 0,003 | 0,0015 | 0,0008 | 0,0004 | 0 |  |
| **E** |  | 0,1 | 0,05 | 0,02 | 0,01 | 0,006 | 0,003 | 0,0015 | 0,0008 | 0,0004 | 0 |  |
| **F** |  |  |  |  |  |  |  |  |  |  |  |  |
| **G** |  |  |  |  |  |  |  |  |  |  |  |  |
| **H** |  |  |  |  |  |  |  |  |  |  |  |  |
