# Supplementary material for: A Nanobody/Monoclonal Antibody “hybrid” sandwich technology offers an improved immunoassay strategy for detection of African trypanosome infections
Source: PLoS Negl Trop Dis. 2024 Jul 1;18(7):e0012294. doi: 10.1371/journal.pntd.0012294 (PMC11244815; doi:10.1371/journal.pntd.0012294)
Supplement: S4 Table — (DOCX) [file pntd.0012294.s010.docx]

**S4 Table.** **A dot blot layout of a Gold-labelled IgM8A2 direct binding to *Tco*ALD. (1)** Spotting onto the nitrocellulose membrane of the recombinant *Trypanosoma congolense* aldolase (*Tco*ALD), *T. congolense* Lysate (*Tco*Lys), *Leishmania mexicana* aldolase (*Lm*ALD), *T. evansi* enolase (*Tev*ENO), or 1xPBS. **(2)** Blotted nitrocellulose membrane immersed in 5% milk solution. **(3)** Spotting of the Gold-labelled IgM8A2 (IgM8A2-G) on each of the sites previously spotted with proteins or 1xPBS.

| **1.** Spot | *Tco*ALD | *Tco*Lys | *Lm*ALD | *Tev*ENO | 1xPBS |
| --- | --- | --- | --- | --- | --- |
| **2.** Blocking | 5% milk solution | | | | |
| **3.** Spot | IgM8A2-G | IgM8A2-G | IgM8A2-G | IgM8A2-G | IgM8A2-G |
