# Supplementary material for: A Nanobody/Monoclonal Antibody “hybrid” sandwich technology offers an improved immunoassay strategy for detection of African trypanosome infections
Source: PLoS Negl Trop Dis. 2024 Jul 1;18(7):e0012294. doi: 10.1371/journal.pntd.0012294 (PMC11244815; doi:10.1371/journal.pntd.0012294)
Supplement: S5 Table — (DOCX) [file pntd.0012294.s011.docx]

**S5 Table.** **A dot blot layout of Nb474H/Gold-labelled IgM8A2 sandwich binding to *Tco*ALD. (1)** Nb474H was blotted at five spots on a nitrocellulose membrane. **(2)** The blotted nitrocellulose membrane was immersed in 5% milk solution. **(3)** The *Trypanosoma congolense* aldolase (*Tco*ALD), *T. congolense* Lysate (*Tco*Lys), *Leishmania mexicana* aldolase (*Lm*ALD), *T. evansi* enolase (*Tev*ENO), or 1xPBS was spotted on each of the sites previously blotted with Nb474H. **(4)** The Gold-labelled IgM8A2 (IgM8A2-G) was spotted on each of the sites previously blotted with Nb474H followed by the proteins (*Tco*ALD, *Tco*Lys, *Lm*ALD, *Tev*ENO), or 1xPBS.

| **1.** Spot | Nb474H | Nb474H | Nb474H | Nb474H | Nb474H |
| --- | --- | --- | --- | --- | --- |
| **2.** Blocking | Block with 5% milk | | | | |
| **3.** Spot | *Tco*ALD | *Tco*Lys | *Lm*ALD | *Tev*ENO | PBS only |
| **4.** Spot | IgM8A2-G | IgM8A2-G | IgM8A2-G | IgM8A2-G | IgM8A2-G |
